# Multi-scenario land use change simulation and spatial-temporal evolution of carbon storage in the Yangtze River Delta region based on the PLUS-InVEST model

**DOI:** 10.1371/journal.pone.0316255

**Published:** 2025-01-24

**Authors:** Jingru Zhou, Verner Carl Johnson, Jingchao Shi, Mou Leong Tan, Fei Zhang

**Affiliations:** 1 College of Geography and Environmental Sciences, Zhejiang Normal University, Jinhua, China; 2 Department of Physical and Environmental Sciences, Colorado Mesa University, Grand Junction, CO, United States of America; 3 Departments of Earth Sciences, the University of Memphis, Memphis, TN, United States of America; 4 Geo-Informatic Unit, Geography Section, School of Humanities, University Sains Malaysia, Penang, Malaysia; Tongji University School of Economics and Management, CHINA

## Abstract

Influenced by urban expansion, population growth, and various socio-economic activities, land use in the Yangtze River Delta (YRD) area has undergone prominent changes. Modifications in land use have resulted in adjustments to ecological structures, leading to subsequent fluctuations in carbon storage. This study focuses on YRD region and analyzes the characteristics of land use changes in the area using land use data from 2000 to 2020, with a 10-year interval. Utilizing InVEST Model’s Carbon Storage module in combination with PLUS model (patch-generating land use simulation), we simulated and projected future land use patterns and carbon storage across YRD region under five scenarios including natural development (ND), urban development (UD), ecological protection (EP), cropland protection (CP), and balanced development (BD). Upon comparing carbon storage levels predicted for 2030 under the five scenarios with those in 2020, carbon stocks decrease in the initial four scenarios and then increase in the fifth scenario. In the initial four declining scenarios, CP scenario had the least reduction in carbon storage, followed by EP scenario. The implementation of policies aimed at safeguarding cropland and preserving ecological integrity can efficaciously curtail the expansion of developed land into woodland and cropland, enhance the structure of land use, and mitigate the loss of carbon storage.

## 1 Introduction

Land is a limited natural resource on Earth, and it is of great importance in agriculture, ecosystem support, city planning, and the protection of ecosystems. Sensible land use and conservation practices can achieve sustainable development and promote a harmonious relationship between humans and nature [1]. Land use/cover change (LUCC) has significant global implications, influencing the geographic distribution of ecosystem services, altering ecosystem patterns, and processes, which in turn affects ecosystem functions [2–4]. In recent years, LUCC and its impact on environmental, economic, and social sustainability have become widely researched topics globally [5–7]. Many land types with high ecological value, such as grasslands, forests, and water bodies, are experiencing a sharp decline in biodiversity due to frequent human activities like urbanization and land development [8]. LUCC is also recognized as a key factor influencing regional carbon storage [9, 10].

In response to global climate change, the Chinese government has committed to achieving peak carbon emissions by 2030 and striving for carbon neutrality by 2060, as outlined in the 14th Five-Year Plan. This aligns with the goals of the Paris Agreement, which aims to keep the rise in global temperature well below 2°C above pre-industrial levels, with efforts to limit it to 1.5°C. Achieving this goal involves reducing greenhouse gas emissions and enhancing carbon sequestration, making land-use practices and LUCC essential drivers of these efforts [11]. LUCC is the most immediate manifestation of human activities that alter the carbon cycling process of vegetation and soil in terrestrial ecosystems, impacting regional carbon stocks [12, 13]. Moreover, LUCC-induced land conversion results in increased atmospheric CO_2_ levels [14, 15].

With the intensification of global climate change and environmental degradation, effectively addressing carbon emissions and increasing carbon storage has become a global focus. Especially after China’s "Carbon Peak and Carbon Neutrality Policy", how to achieve these goals while ensuring the sustainable development of regional economies has become a critical issue. LUCC is an important component of the global carbon cycle, and the transformation and development of land directly affect carbon storage and ecosystem services. Optimizing land use and its layout is considered one of the most cost-effective strategies for carbon sequestration [16]. By promoting sustainable and low-carbon development and establishing a robust "dual-carbon" policy framework, significant progress can be made toward a sustainable and carbon-neutral future [17, 18]. This underscores the growing importance of assessing carbon stocks across various spatial-temporal scales. Woodland resources are crucial in carbon absorption, and large-scale deforestation and land conversion reduce carbon sinks, thus hindering carbon neutrality goals [19]. Carbon sequestration potential varies across different LUCC types, and the spatial dynamics of LUCC influence plant growth, soil composition, and ecosystem carbon stocks [20]. A study on LUCC dynamics in China’s terrestrial ecosystems between 1980–2010 showed a depletion of 279 Tg of carbon stock [21, 22]. Moreover, socio-economic factors [23] such as population growth [24], economic development [25], policy changes [26], and market demand [27] increasingly shape LUCC patterns. Machine learning technologies, including deep learning and reinforcement learning, are becoming important tools in land-use modeling [28, 29], helping to uncover complex nonlinear relationships from large datasets and better capture LUCC patterns. The integration of socio-economic factors, big data, and machine learning enhances model accuracy and predictive power, contributing to advances in LUCC research [30, 31].

At present, common land use simulation models include CLUS [32], FLUS [33], SD [34], CA-Markov [35], PLUS [36], etc., among which PLUS model, being superior in simulating fine patches, utilizing an adaptive inertia competition mechanism to calculate the integrated probability of land use change [37]. It is particularly effective in areas with fine patches and multiple LUCC types, providing a detailed representation of LUCC dynamics. Many studies have successfully used the PLUS model for land-use analysis [37–39]. Alongside field surveys [40], computational models like the CENTURY [41], DSSAT [42], and COMET-Farm [43] are increasingly used to assess carbon stocks and predict carbon cycling. The Global Carbon Project (GCP) [44] provides comprehensive data and models for global carbon emissions and absorption, integrating remote sensing and ground observation data to provide a more accurate global carbon budget. The Carnegie-Ames-Stanford Approach (CASA) [45] simulates the carbon fixation capacity of ecosystems, focusing on plant photosynthesis and respiration. The InVEST model [46–48], known for its precision and efficiency, has been widely applied for carbon storage estimation. In summary, the combination of the PLUS and InVEST models is highly effective for simulating complex LUCC processes and estimating carbon stocks [49, 50]. This study aims to use these models to analyze the dynamics of land use and carbon stocks in the Yangtze River Delta (YRD) region, across various development scenarios. The novelty of this study lies in the inclusion of multiple scenarios and the assessment of their compatibility with China’s “dual carbon policy,” offering a comprehensive perspective on future land-use planning and development in the region. This study will focus on how land-use transformations influence ecosystem carbon storage, a critical factor in safeguarding ecosystems and promoting sustainable development in the YRD region [51]. As one of China’s most economically developed regions, the YRD plays a key role in achieving the country’s dual-carbon goals [52].

In recent years, China’s rapid economic growth, accelerated urbanization, and agricultural expansion have altered land use patterns to varying degrees, subsequently affecting regional carbon storage capacity. However, research on how these land use changes specifically affect carbon storage remains insufficient, particularly in the rapidly developing YRD region, where the dynamic relationship between LUCC and carbon storage is still unclear. Therefore, conducting this study is of significant importance as it not only helps assess the impact of LUCC on regional carbon storage, but also provides scientific evidence for policymakers, supporting regional sustainable development and the achievement of carbon neutrality goals [15, 53, 54]. YRD has seen rapid economic growth, urbanization, and industrialization, resulting in significant land-use changes. The Outline of the Plan for the Integrated Development of the YRD Region (2019) emphasizes enhancing ecological protection and collaborative governance. It aims to create an ecologically friendly development model for the region and build a demonstration area for integrated, ecologically sustainable development at a high level [55]. Understanding land-use changes in the YRD under different future scenarios, and their impact on carbon stocks, is crucial for planning a sustainable future for this region.

The objectives of this study are to: (1) examine the historical and current land use changes at 10-year intervals, (2) integrate PLUS-InVEST model to project land use and carbon stock maps for 2030 under five different scenarios, and (3) assess the impact of land-use changes on carbon stocks from 2000 to 2030. The unique contribution of this study is its adoption of a multi-scenario carbon stock assessment methodology, which is closely aligned with the objectives of China’s ’dual carbon policy’. The study provides an in-depth analysis of YRD region, offering region-specific insights into ecological and economic activities. By exploring multiple scenarios, including ecological protection and cropland conservation, this study presents a comprehensive view of future carbon storage dynamics. It also emphasizes the need for prioritizing carbon storage in land-use planning and policy development, which is essential for long-term environmental sustainability. Scenario simulations will help evaluate the impact of policy decisions on carbon storage and provide valuable insights for balancing economic growth with ecological protection. This study is aligned with China’s emission reduction goals under the Paris Agreement, contributing to monitoring and evaluating progress towards carbon neutrality.

## 2 Materials and datum

### 2.1 Study area

**Fig 1:**
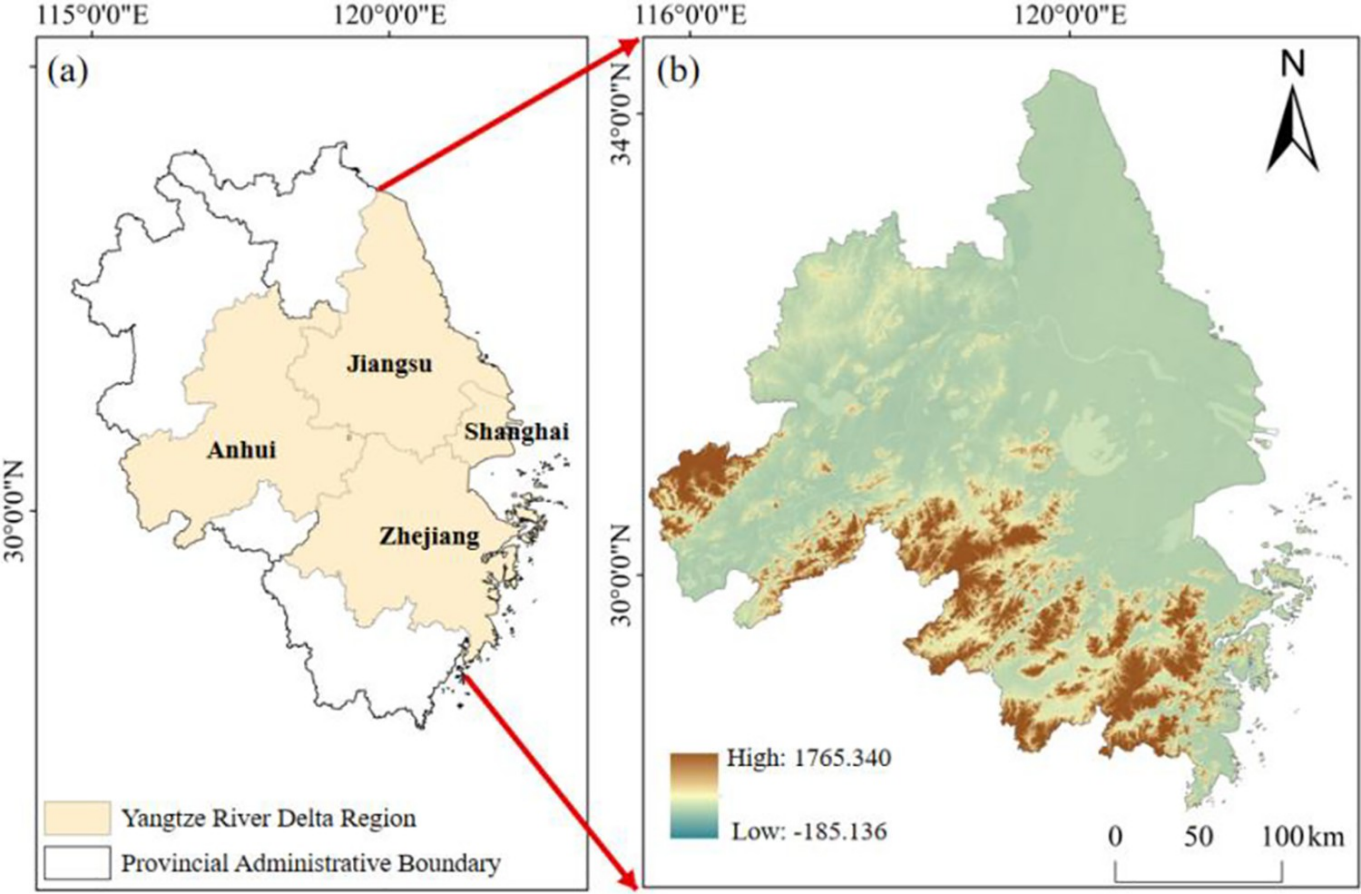
Geographic location of research area ((a). Geographic location of YRD (Basemap Sources:https://www.tianditu.gov.cn/, Revision No.:GS(2024)0650); (b). The elevation of the target area).

YRD in China is known worldwide as the largest cluster of neighboring metropolitan areas [56]. Geographically, it is situated between longitude 116°00’ to 124°30’ E and latitude 28°30’ to 34°30’ N. Shanghai, Jiangsu, Zhejiang, and Anhui are constituent regions of this area (Fig 1). YRD region holds significant importance as an economic center in China, serving as one of country’s most significant industrial bases and economic hubs [57]. Approximately 10% of the country’s total economic yield is contributed by the region in question. It is supported by high-tech manufacturing and service industries. Within YRD region, it has evolved into a globally significant hub for advanced manufacturing and serves as a prominent world portal within the Asia-Pacific region, and China’s largest economic zone [58]. It is also one of China’s important agricultural regions, with major crops such as rice, wheat, corn and other crops. YRD region features a subtropical monsoon climate, which is distinguished by mild and humid weather conditions, distinct four seasons, and abundant rainfall. Characterized by a moderate climate, the region has an average annual temperature between 15 to 18°C.

### 2.2 Data sources and processing

#### 2.2.1 LUCC data

With a favorable geographical location and diverse land use types, YRD region encompasses 26 cities [59]. Land use data for YRD region (2000–2020) is sourced from GlobalLand30 (http://www.globallandcover.com). By using ArcGIS for mosaicking, masking, and cropping, study area images are obtained. Considering the actual conditions within YRD region, land use has been categorized into six distinct types, including watershed, woodland, grassland, cropland, developed land, as well as unused land. This classification was done using the Reclassify tool in ArcGIS 10.6.

#### 2.2.2 Driving factors

**Fig 2:**
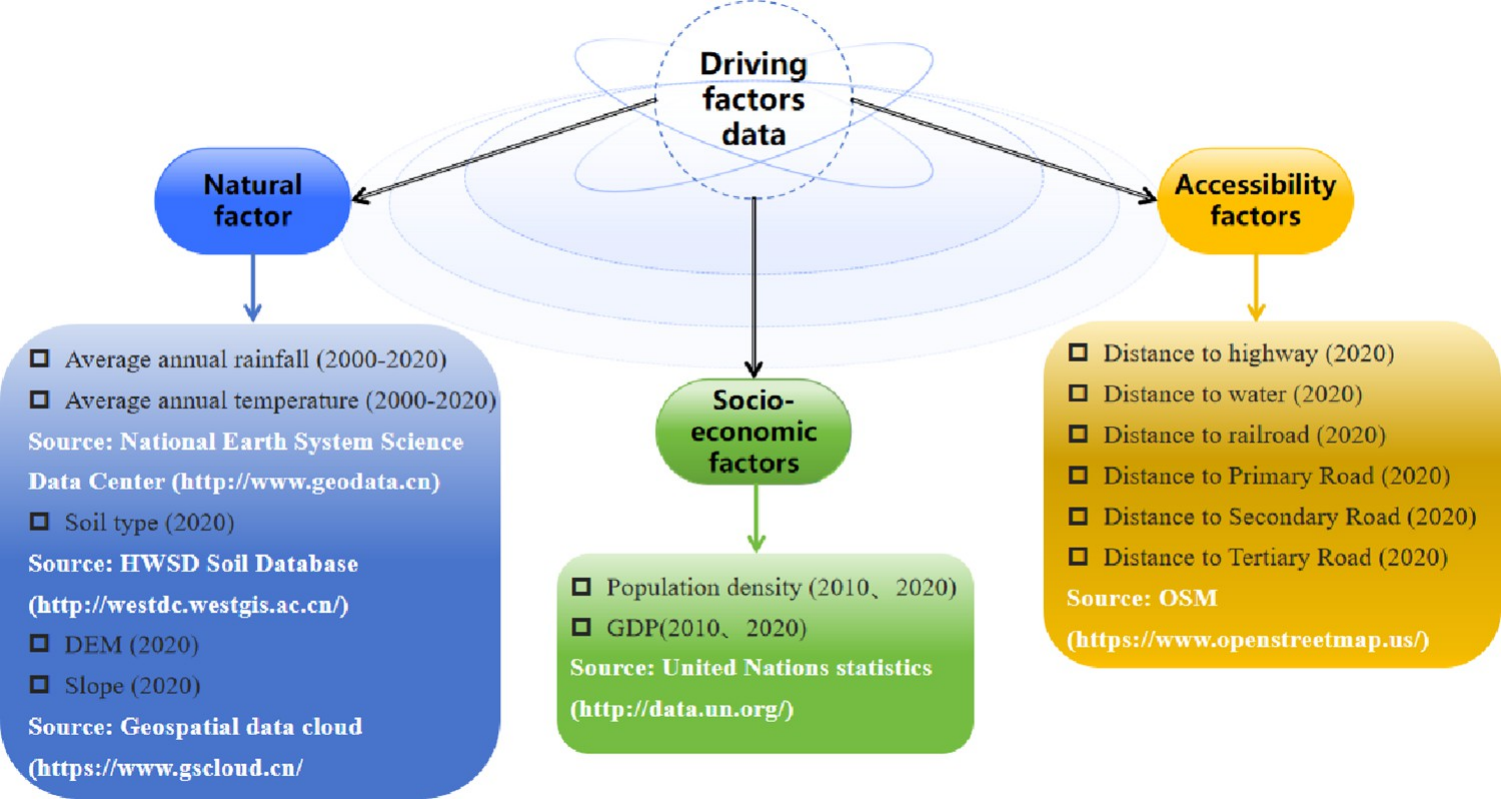
Details regarding the data utilized.

In this study, 13 driving factors were employed to analyze land use changes in YRD region. These factors encompassed population density, per capita GDP, annual precipitation, average temperature, DEM, slope, distance to primary, secondary, and tertiary roads, distance to water, distance to railroad, and soil types. These factors were categorized into three main types: socio-economic, natural, and accessibility factors (Fig 2). Vector data were converted into raster format using Euclidean distance in ArcGIS, with a spatial resolution set at 30m.

Population density has a direct impact on urban expansion and the demand for developed land. In the ND and UD scenarios, densely populated areas may drive the expansion of urban and residential areas. However, in the CP or EP scenarios, development in these areas may be limited to prevent the overexploitation of natural resources and cropland. High per capita GDP is typically associated with a higher level of urbanization and infrastructure development. In the UD scenario, regions with high per capita GDP will likely attract more developed land, facilitating the expansion of commercial and industrial areas. In contrast, in the EP scenario, land use patterns may prioritize the preservation of ecosystem health, even if GDP is high.

Climate variables also play a significant role in determining land use suitability. Regions with high rainfall and moderate temperatures are typically suitable for cropland, woodland or wetlands. Therefore, in the CP and EP scenarios, these areas may be prioritized for agricultural or ecological use, while in the ND scenario, land use changes in these areas might be more pronounced. Topographical factors directly affect the feasibility of land development, where steep slopes are typically unsuitable for large-scale urban construction and are often preserved as natural areas in EP scenario. In contrast, flat terrains are more conducive to land expansion in the UD scenario.

Infrastructure accessibility is a major factor driving key urban expansion. Areas near major roads, particularly in the UD and ND scenarios, are often prioritized for development. However, in the CP or EP scenarios, even with high accessibility, these areas may still be preserved as agricultural or natural land. Water sources are vital for ecological protection, and regions near these sources may be prioritized for conservation in the EP scenario to maintain wetlands and aquatic ecosystems. In the UD scenario, the use of these areas might be more restricted. Additionally, railways contribute to urban and industrial expansion. Areas closer to railways are more likely to be developed land, whereas the EP or CP scenarios, development in these regions may be ore regulated.

In the ND scenario, socio-economic and accessibility factors may drive urbanization larger, while the influence of natural factors diminishes, leading in the overexploitation of natural resources. The CP scenario prioritizes the protection of high-quality cropland, where natural factors such as rainfall and soil types are crucial, particularly in areas suitable for agriculture. In this scenario, these regions receive priority for conservation. In contrast, natural factors such as water sources, climate, and topography play a dominant role in the EP scenario, where the protection of key ecological functional areas, including wetlands, woodland and water sources, is prioritized. In the UD scenario, accessibility factors such as proximity to roads and railways dominate, driving the expansion of urban and industrial land, which may compromise the protection of natural and agricultural areas. The BD scenario aims to balance socio-economic development with the protection of natural resources by considering various factors to promote orderly urban expansion and sustainable resource use. Current simulation models primarily center on the changes of individual land types resulting from human activities, often with limited consideration of policy interventions in land supply. To address this gap, the PLUS model integrates socio-economic, natural, and accessibility factors to simulate various development scenarios of future land types.

#### 2.2.3 Carbon intensity data

Carbon intensity data used in current study for YRD region is sourced from published literature [50]. Building upon previous research [60], carbon density of rivers was disregarded [61], then the data was adjusted based on the findings [62]. Carbon density values for six land use categories in YRD region were determined using carbon stock conversion formulas [54], as shown Table 1 [63].

**Table 1:** Carbon intensity data for YRD region Unit:kg/m^2^.

LUCC type	C_above_	C_below_	C_soil_	C_dead_
Woodland	1.8873	1.2457	8.6759	0.2410
Cropland	3.6339	0.7268	12.0758	0.3354
Grassland	1.7374	2.0849	10.5847	0.2940
Developed land	0.0000	0.0000	8.1100	0.0000
Watershed	1.6153	0.3231	7.2920	0.0000
Unused land	2.4291	0.4858	8.0719	0.2242

## 3 Method

### 3.1 Land use dynamics

Land use dynamics provide an effective representation of changes in land resource quantity [64]. Additionally, single land use dynamics can capture the specific changes of a particular type within the scope of the study over a defined time frame [65]. Formula is shown in Eq (1).


$$K=\frac{U_{b}-U_{a}}{U_{a}}\times \frac{1}{T}\times 100\%$$ (1)

Where *K* represents the single dynamic index of land use; *U*_*a*_ and *U*_*b*_ respectively represent the original and final areas of land use types in research area; and *T* is time frame.

Comprehensive land use dynamics serves as an indicator of the pace, at which land use changes occur in research area. The formula is shown in Eq (2).


$$R_{\mathrm{total}}=\frac{\Sigma_{i=1}^{n}\Delta LU_{i}}{2\Sigma_{i=1}^{n}LU_{i}}\times \frac{1}{T}\times 100\%$$ (2)

Where *LU*_*i*_ represent the area of the *i*-th land use type converting into other types at the commencement of the current research; *ΔLU*_*i*_ represent the area of the *i*-th type converting into other types; *T* represent study period.

### 3.2 Land use transition matrix

Land-use transformation matrix can accurately reflect the direction and extent of land-use transformation in study area over a given period of time [64]. It provides a systematic and quantitative description of changes in land use, visually presenting the direction and quantity of land transfers. To fully reveal transformation features of land use types in YRD region between 2000 and 2020, a land use transition matrix was built based on a 10-year cycle. This matrix provides a land use status map and relevant data, enabling an analysis of land use change patterns in YRD region.

### 3.3 Land use simulation

PLUS model effectively addresses the limitations observed in existing simulation models [37], particularly in simulating diverse patch scales. Land use simulation data from PLUS model comprises land use type data, restricted conversion area data, and driver data. Specifically, land use type data include remote sensing monitoring information for the years 2000 and 2020. A total of 13 drivers, in terms of nature, society and accessibility, are selected. It is ensured that the rasterized driver data and land use type data have consistent projection coordinates and spatial resolution. Secondly, LEAS module is used to simulate land use expansion from 2010 to 2020 to get the probability of development of each type of land use in YRD region. Then, combining the future land use demand of each category under different scenarios, domain weights and transfer matrices and other related parameters, CA model based on multi-class stochastic patch seeding is simulated to predict land use changes in YRD region in 2030. Finally, 2020 land use type data simulated by PLUS model is compared with actual 2020 land use type data for validation. In PLUS model, socioeconomic data, such as population and economic activity, are used as input variables, along with defined land use conversion rules. The model computes and predicts land use patterns and changing trends under various scenarios. Different socioeconomic driving factors significantly affect land use change scenarios by influencing the spatial distribution and conversion patterns of land use. Relevant parameters are set as follows:

1. Land use demand
  Land use demand refers to the quantity of raster cells for different land use types in the simulation year. This demand is primarily estimated using two methods: Linear regression and Markov chain. In our study, Markov chain method is employed to forecast the number of raster cells for each land use type in 2030.
2. Area weights
  Domain weights indicate the expansion capacity of different land use types, and the amount of change in the area (TA) of each land use type in the same time scale better reflects its expansion capacity. In this study, the dimensionless value of *ΔTA* from 2010 to 2020 was used to determine the domain weights (Table 2), and calculation formula is shown in Eq (3).
$$w_{i}=\frac{\Delta TA_{i}-\Delta TA_{\min}}{\Delta TA_{\max}-\Delta TA_{\min}}$$ (3)
  Where: *ΔTA*_*i*_ represents the change in the expansion area for each type; *ΔTA*_*min*_ denotes the minimum change in expansion area for each type; *ΔTA*_*max*_ indicates the maximum change in expansion area for each type.
3. Transfer matrix
  Transfer matrix illustrates rules for converting between various land use types. The specific rule is that when one land use type cannot be converted to another land use type, the corresponding value in the matrix is 0, and vice versa is 1. Since two land use types are not bordering, they cannot be directly converted in general. The transition matrix in this paper is shown in Table 3.

**Table 2:** Neighborhood weights for different land use types simulated by PLUS model.

LUCC type	Woodland	Cropland	Grassland	Developed land	Watershed	Unused land
Area weights	0.008052	1.000000	0.004076	0.911642	0.195485	0.000001

**Table 3:** Transfer cost matrix of each land use type under different development scenarios.

Development scenarios		Cropland	Woodland	Grassland	Watershed	Developed land	Unused land
ND	Cropland	1	1	1	1	1	1
	Woodland	0	1	1	1	1	1
	Grassland	1	1	1	1	1	1
	Watershed	1	1	1	1	1	1
	Developed land	1	0	0	1	1	1
	Unused land	1	0	1	1	1	1
UD	Cropland	1	1	1	1	0	1
	Woodland	1	1	1	1	0	0
	Grassland	1	1	1	1	0	1
	Watershed	1	0	1	1	0	1
	Developed land	1	1	1	1	1	1
	Unused land	0	0	1	1	0	1
EP	Cropland	1	0	0	0	1	1
	Woodland	1	1	1	1	1	1
	Grassland	1	0	1	1	1	1
	Watershed	1	0	0	1	1	1
	Developed land	1	0	0	0	1	1
	Unused land	0	0	0	0	1	1
CP	Cropland	1	0	0	0	0	0
	Woodland	0	1	1	0	1	1
	Grassland	0	1	1	1	1	1
	Watershed	0	0	1	1	0	1
	Developed land	0	1	1	1	1	1
	Unused land	0	1	1	1	1	1
BD	Cropland	1	1	1	1	1	1
	Woodland	0	1	1	1	1	1
	Grassland	1	1	1	1	1	1
	Watershed	1	1	1	1	1	1
	Developed land	1	0	0	1	1	1
	Unused land	1	0	1	1	1	1

### 3.4 Multiple scenarios for land use prediction

"National Master Plan for Main Functional Zones," "National Master Plan for Main Marine Functional Zones," and "Outline of Integrated Development Plan for YRD Region" have put forward inherent requirements for the construction of mountains, rivers, woodland, cropland, lakes, cities, and villages, as well as the realization of ecological protection. Drawing upon aforementioned plans, current research examines the impacts of diverse land development strategies on land use patterns. Furthermore, it examines the space layout and structural arrangement of land use in YRD region, which take into account different scenarios, such as ND, UD, EP, CP and BD scenario, for the year 2030.

By incorporating Markov chain module of PLUS model, the forecasted land use demand for 2030 is derived under the ND scenario, considering the historical trend of land use changes between 2010 and 2020. By using Markov chain to anticipate land demand, based on similar study areas and the findings of scholars [66]. EP scenario is to protect areas of woodland and grassland, which can be more easily converted to woodland and grassland, except for construction land. Under the EP scenario, transfer probabilities for land use conversion are modified, with a 50% reduction for woodland and grassland being turned to developed land, a 30% reduction for cropland being converted to developed land, and a 30% increase for cropland being turned to woodland and grassland. Under the UD scenario, land use transfer probabilities are set to increase by 20% for the conversion from cropland, grassland, and woodland to developed land, and decrease by 20% for the conversion from developed land to land uses except apart from cropland. Cropland is an important basis for consolidating and improving food production capacity and guaranteeing national food security, and the scope of existing cultivated land is strictly controlled. Its quality is focused on improving in accordance with the optimal protection model. Under the CP scenario, land use transfer probabilities are set to reduce by 20% for the conversion from woodland and grassland to developed land and to drop by 60% for the conversion from cropland to developed land. The objective of BD scenario is to protect ecology based on protecting cropland, which is of great significance to YRD region and is related to food security, ecological environment and sustainable development. This scenario reduces the transfer probability of shifting cropland, woodland, grassland and water to developed land by 20% and increases the probability of shifting developed land to cropland, woodland and grassland by 30%, and involves no policy interventions, and there are no restrictions on shifting between different land categories. Cost matrix in the multiple scenarios only allows developed land to remain unchanged. Using these configurations, RF algorithm is employed to determine land use categories.

### 3.5 Carbon storage estimation

To evaluate the time-space variation in carbon storage within YRD region between 2000 and 2030, Carbon Storage module was utilized. This module incorporates above-ground biomass carbon density, below-ground biomass carbon density, soil carbon density, and dead organic carbon density for each LUCC type [46]. Spatial distribution of carbon stocks was estimated by combining LUCC data and its corresponding carbon pool data. Specific Eqs are as follows (4) ~ (5):

$$C_{i}=C_{i-above}+C_{i-below}+C_{i-soil}+C_{i-dead}$$ (4)

$$C_{\mathrm{totali}}=\sum_{i=1}^{n}C_{i}\times A_{i}$$ (5)

where *i* is a specific land use type; *C*_*i*_ is carbon density of land use type *i*; *C*_*i-above*_, *C*_*i-below*_, *C*_*i-soil*_ and *C*_*i-dead*_ are above-ground vegetation carbon density (t∙hm^-2^), below-ground vegetation carbon density (t∙hm^-2^), soil carbon density (t∙hm^-2^) and dead organic carbon density (t∙hm^-2^), respectively, for type *i*. *C*_*total*_ is total carbon stock of ecosystem (t), and *A*_*i*_ is area (hm^-2^) of land use type of category *i*, and n is the number of land use types. Carbon density data were calculated using the average annual temperature plus its precipitation in and around study area, adjusted using a correction formula.

**Fig 3:**
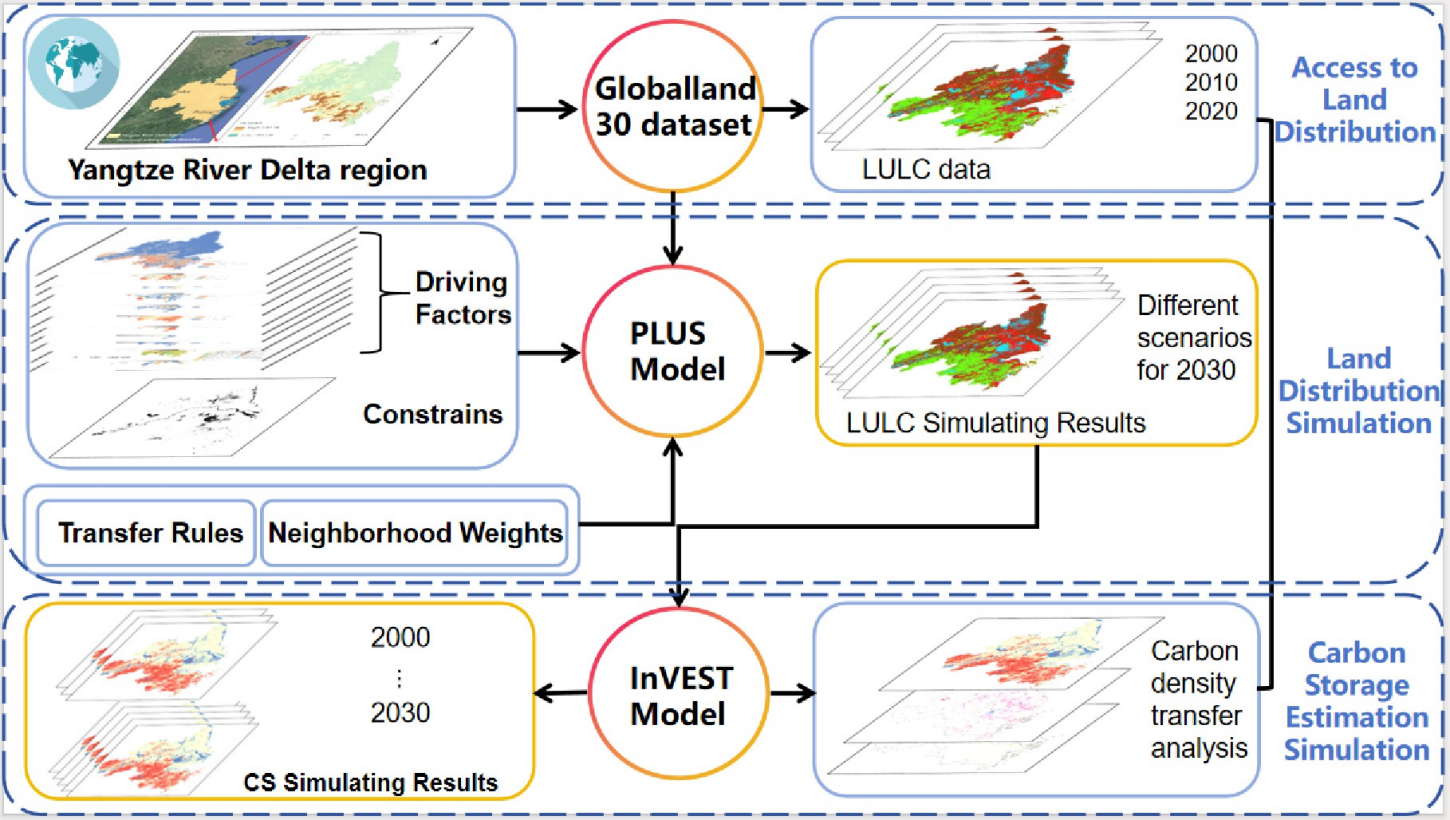
Flowchart of methodology in our study.

Process framework of this research is shown in Fig 3.

## 4 Results and analysis

### 4.1 Analysis of LUCC

#### 4.1.1 Spatial-temporal distribution

**Fig 4:**
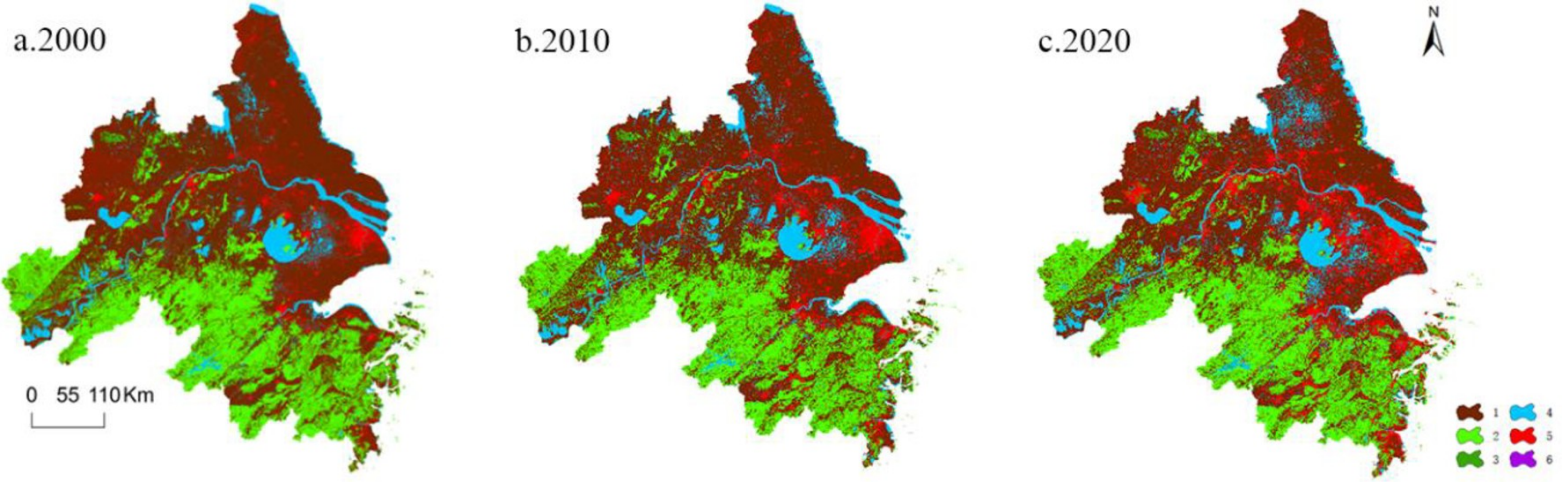
Categorization of land use (1-cropland, 2-woodland, 3-grassland, 4-watershed, 5-developed land, 6-unused land).

Spatial distribution of land use categories in YRD region between 2000 and 2020 is illustrated in Fig 4. Woodland is predominantly situated in the southern part of Yangtze River, while cropland is primarily distributed in the northern area. Woodland and cropland constitute a significant proportion of land cover. Table 4 depicts dynamics of each land use category based on Formulas (1) and (2). Between 2000 and 2020, urbanization of YRD region has changed significantly in developed land, showing a decreasing trend from east to west. The order of change speed for other land cover types goes in order unused land > cropland > watershed > grassland > woodland. Upon analyzing temporal dynamics of land use, a clear trend emerges between 2000 and 2020. Grassland, watershed, developed land, and unused land have exhibited an increase, while cropland and woodland have experienced a decline. To fulfill the requirements of economic growth and urbanization, land resources in some areas have been exploited through activities such as mining, land leveling, and water management projects, leading to an increase in unused land area.

**Table 4:** Attitude of land use in YRD region.

Type			2000–2010	2010–2020	2000–2020
Single Dynamic of Land Use	Cropland	-0.48		-1.01	-0.72
	Woodland	-0.10		0.01	-0.05
	Grassland	0.45		-0.11	0.17
	Watershed	0.30		0.53	0.43
	Developed land	4.68		5.28	6.22
	Unused land	3.57		65.18	46.00
Comprehensive Dynamic of Land Use		0.31		0.53	0.42

It will be inferred that LUCC between 2010 and 2020 surpasses the change observed from 2000 to 2010. This phenomenon can be attributed to the influence of rapid economic growth and policy interventions within YRD region.

#### 4.1.2 Analysis of drivers of LUCC

**Fig 5:**
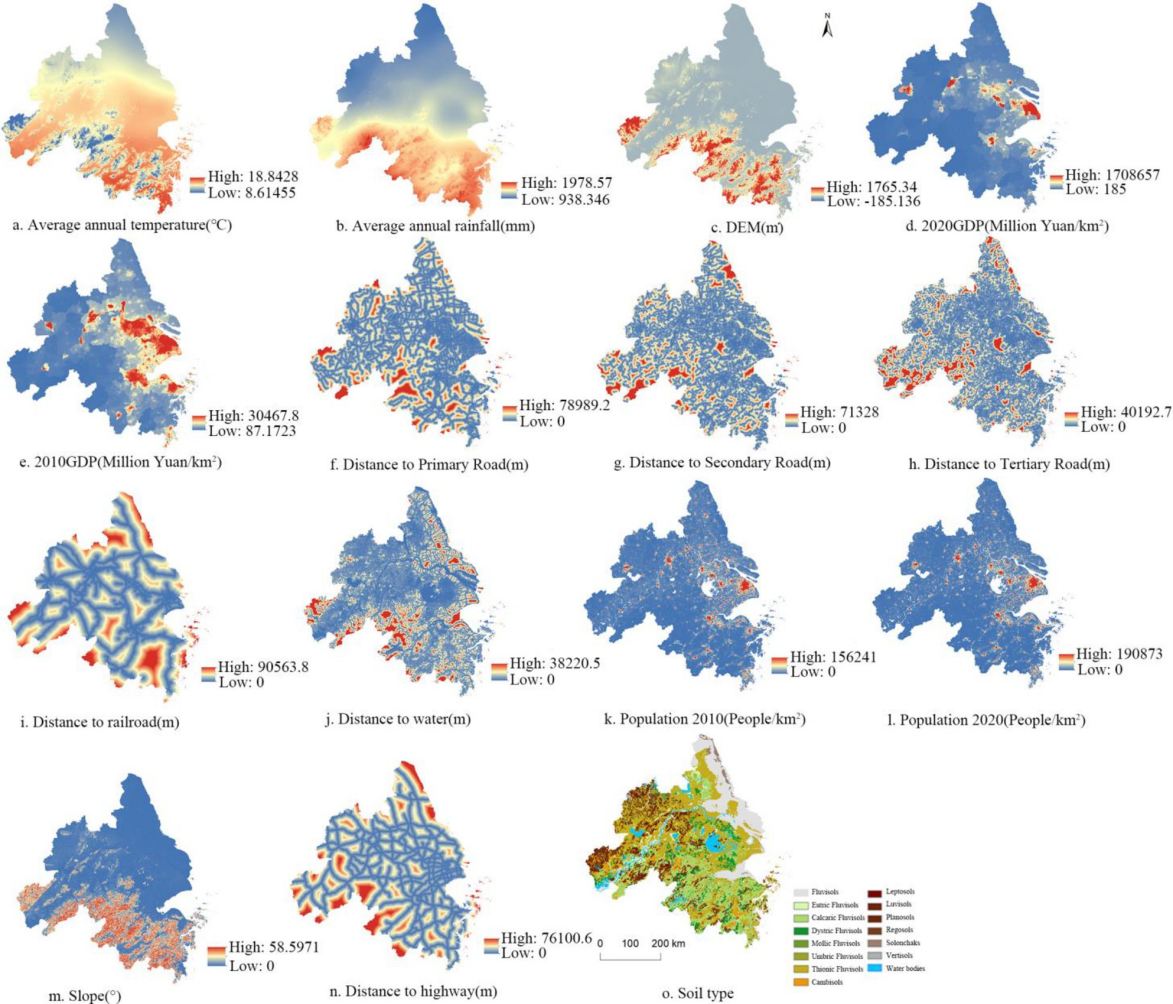
Drivers of urban sprawl in YRD region.

**Fig 6:**
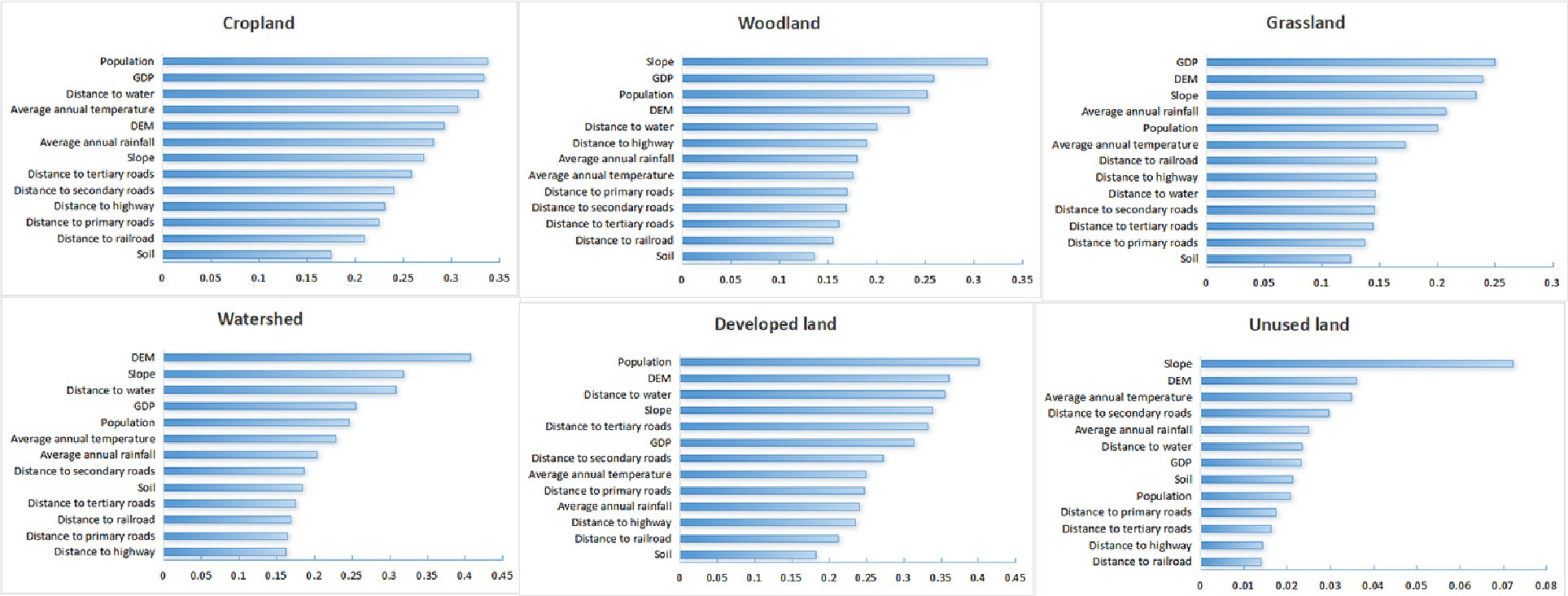
Contribution of drivers.

PLUS model was used to analyze the drivers of LUCC, utilizing data from 2000, 2010, as well as 2020, along with 13 selected driving factors (Fig 5). Fig 6 reveals that population, GDP, and distance to water are primary driving factors for changes in cropland. Rapid population growth in YRD region has led to an increased demand for housing, infrastructure, and industrial facilities, which has directly contributed to occupation of cropland. Secondly, urbanization has been expanding, occupying a large amount of agricultural land. In response to challenges posed by population growth, governments often pursue land-use planning and urban expansion policies, which typically favor conversion of agricultural land to urban or industrial land, thus exacerbating the decline in cropland. GDP growth reflects increased economic activity, and high GDP regions typically require more land for industrial and commercial facilities, resulting in the transformation of cropland into non-agricultural purposes and speeding up land changes. Local and central governments introduced a series of policies to promote economic growth, such as favorable land policies and tax incentives, which have also contributed to conversion of cropland to some extents. Water resources are essential for agricultural productivity, and the distance of water sources affects the efficiency of the use of cropland. Cropland in YRD region is often concentrated in areas closer to major water sources, which is conducive to irrigation. However, policies on water resource protection and water infrastructure construction in YRD region may result in some adjustments that agricultural land was changed to protected or development zones, further affecting the area of cropland. Government policies, including urban-rural planning, cropland protection, and environmental protection, play a regulatory role in changes to cropland. Socioeconomic, policy, and environmental factors collectively influence the distribution and trends of cropland changes in the region. The growth of developed land is primarily driven by population and DEM. Population growth serve as a key factor in land development, as the population increases, there is a corresponding rise in demand for housing, commercial spaces, and infrastructure, which drives urban expansion and land development.

In the YRD region, high population density has created high demand for urbanization, leading to the conversion of surrounding farmland and vacant land into construction areas. Moreover, in densely populated areas, the economic value of land tends to increase, further stimulating its development and use. High population density areas typically experience higher land values, which in turn drives development activities. Population growth directly increases the demand for additional construction areas, promoting the expansion of developed land. DEM provides essential information about terrain, drainage, and associated risks, helping in planning and determining suitable areas for development. Together, these factors shape the growth patterns of developed land in the YRD region. The increase in developed land is concentrated in areas with high population density and relatively flat terrain. Slope emerges as the primary driving factor for expansion of woodland, followed by GDP, population, and DEM, with a notable concentration of woodland expansion in higher altitude regions. With the increasing global concern for environmental protection, YRD region is also implementing a series of environmental protection policies, including restoration and protection of forest ecosystems. In mountainous areas, as large-scale agricultural farming is not suitable for areas with large slopes, it is easier to convert these slopes into woodland to restore the ecosystems under the impetus of policies, such as returning cropland to forests and greening barren hills. As the economy develops, people’s needs and understanding of natural resources are changing. In certain steep-slope areas, as agricultural production is not efficient, shifting to woodland cultivation or eco-tourism development may bring better economic and social benefits. Thus, these areas with larger slopes may be converted to woodland under the influence of relevant policies and market mechanisms. Prime driving forces behind changes in grassland area are GDP with DEM. GDP growth is usually accompanied by an increase in economic activities, especially urbanization and industrialization. This will increase the demand for land, which will lead to a conversion of grassland to other land uses. To achieve a balance between economic growth and environmental conservation, governments will introduce ecological compensation policies or compensatory measures for the protection of some grassland or for the restoration of ecosystems, but these measures may not be sufficient to counteract impacts of economic development on grasslands in the context of rapid GDP growth. In addition, flat terrain is more suitable for grassland, while steep or high-altitude areas may not be suitable for grassland and will therefore be transformed into other types of land. Main driving factors for changes in watershed are DEM and slope. YRD region is home to many rivers, lakes, and coastlines, including Yangtze River, Taihu Lake, Qiantang River, Hangzhou Bay, etc. DEM provides detailed data on terrain and water flow, enhancing the accuracy of predictions and management regarding changes in watersheds. Slope has a direct impact on erosion, sedimentation, and water flow velocity, all of which significantly influence the shape and evolution of the watershed. Collectively, these factors shape the patterns of watershed change and the development of the water system. The dynamics of unused land are primarily influenced by slope, which directly affects land suitability, erosion risk, water flow management, and development difficulty. These factors determine the distribution and dynamic changes of unused land, with steep slope areas often remaining undeveloped due to their limitations, while flatter areas are more preferable to be developed and utilized.

### 4.2 Modeling and forecasting the distribution

**Fig 7:**
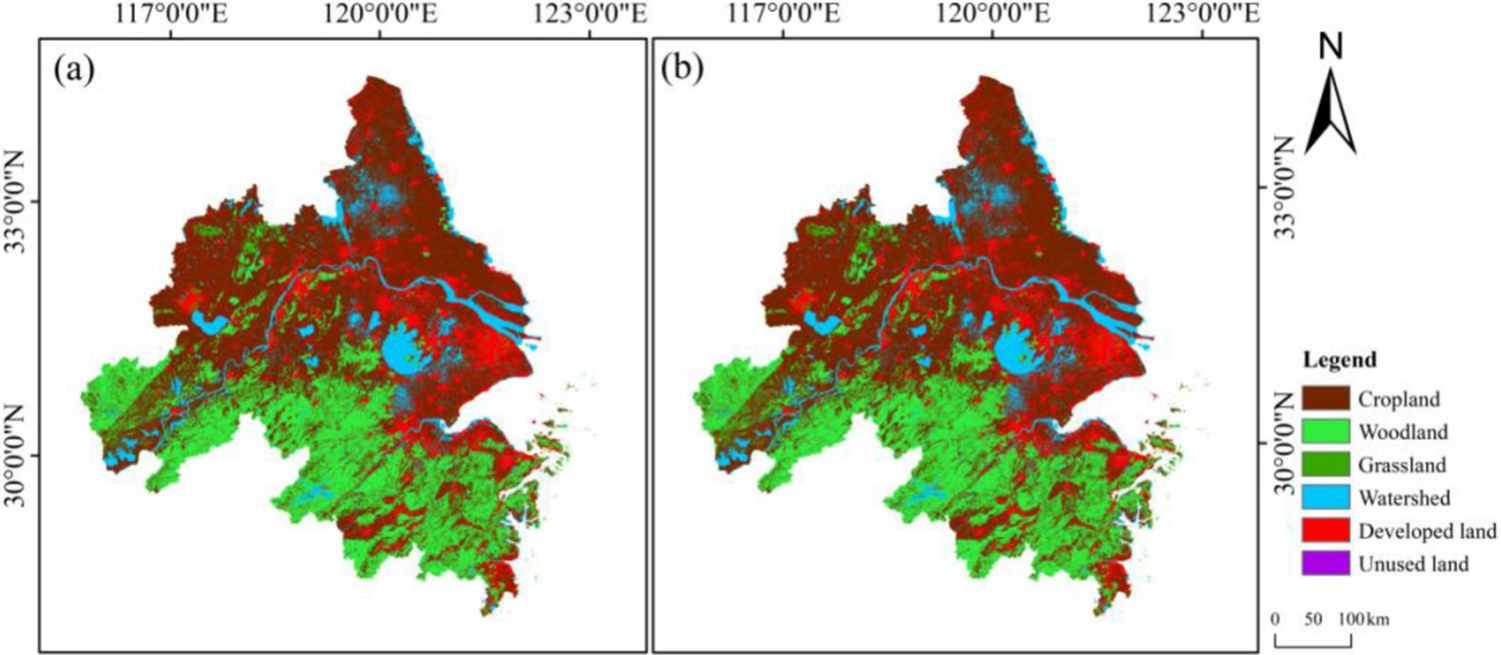
Comparison of land use simulation results and actual land use in YRD in 2020 (a). Simulated land use map of YRD region in 2020; (b) Actual land use map of YRD region in 2020.

**Fig 8:**
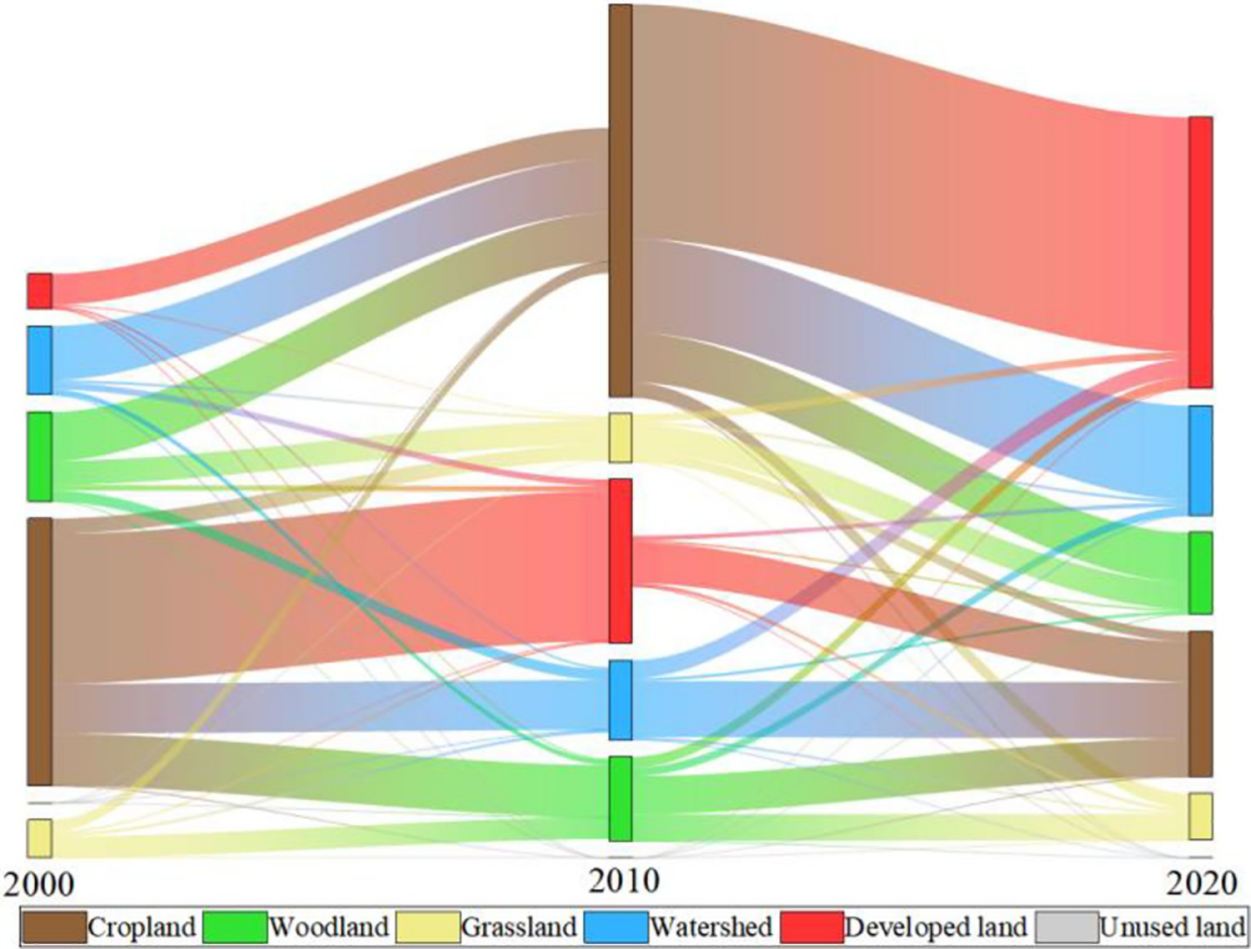
Land-use transfer Sankey diagram.

To evaluate the simulation accuracy of the PLUS model, an analysis of land use expansion strategies was conducted using land use data in 2000 and 2010. The spatial pattern of land use for 2020 was simulated by integrating datasets of selected driving and limiting factors, and the simulation maps were compared with actual maps (Fig 7). The prediction accuracy are presented in Table 5. The findings show that the simulation results align well with the current situation, demonstrating consistency with the overall structure of existing conditions. The Kappa coefficient for the simulated data is 0.74, the FoM value is 0.19, and the overall accuracy is 0.82. These results confirm that the PLUS model can accurately reflect the trends in land-use change and is suitable for simulating future LUCC. As shown in Table 6, during this period, the region of cropland decreased by 17,053.76km^2^, a decrease of 14.44%, while woodland decreased by 535.23 km^2^, a decrease of 0.96%. Grassland increased by 183.33 km^2^, an increase of 3.34%, while watershed rose by 1,629.69 km^2^, an increase of 8.51%, mainly from woodland, cropland and grassland. Due to urbanization, the area of developed land rose the most, by 15,748.51 km^2^, an increase of 124.43%, with most of it coming from cropland and grassland, followed by woodland. The change in unused land was the smallest, increasing by only 27.46 km^2^. From 2000 to 2020, the area of cropland that was converted out was the largest, mainly towards developed land, followed by woodland, indicating that the primary driver behind the enlargement of developed land and woodland was transformation of cropland (Fig 8).

**Table 5:** Comparison of land use prediction accuracy in 2020.

LUCC types	Actual raster	Simulation raster	Same raster	Accuracy	Kappa	Figure of merit (FoM)
Cropland	112284314	112297447	95143001	0.8472	0.74	0.19
Woodland	61064440	61062357	57093121	0.9350		
Grassland	6326152	6321133	632113300	100.0000		
Watershed	23939538	23949749	23221677	0.9696		
Developed land	32159022	32322376	21769120	0.6735		
Unused land	33641	29910	21601	0.7222		

**Table 6:** Area of each category in YRD region Unit:km^2^.

Type	2000	2010	2020
Cropland	118,105.98	112,405.79	101,052.23
Woodland	55,601.35	55,038.53	55,066.13
Grassland	5,496.09	5,741.14	5,679.42
Watershed	19,153.69	19,734.42	20,783.38
Developed land	12,656.26	18,584.31	28,404.77
Unused land	2.98	4.05	30.44

**Fig 9:**
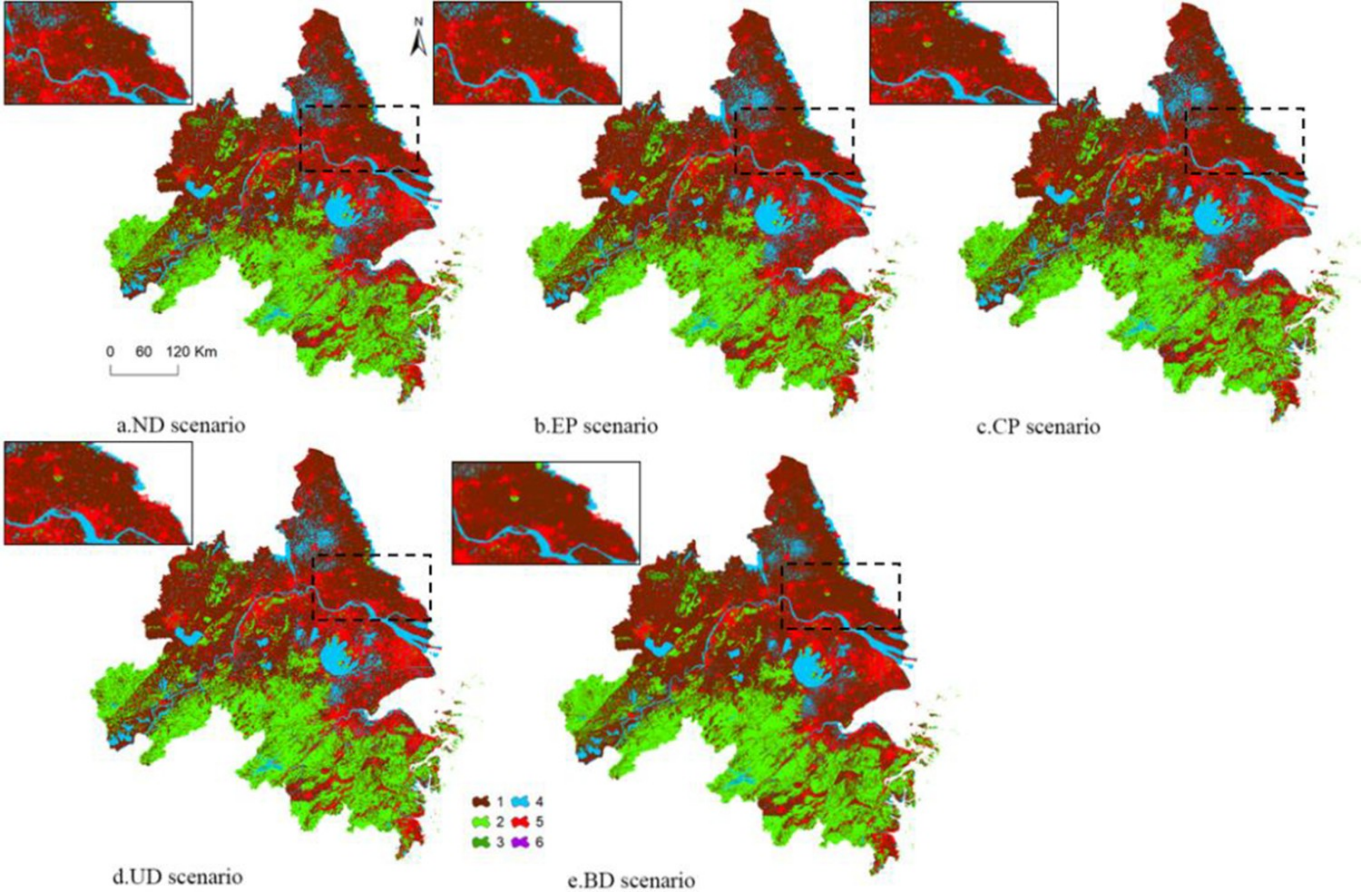
Land use map for 2030 under various scenarios.

**Fig 10:**
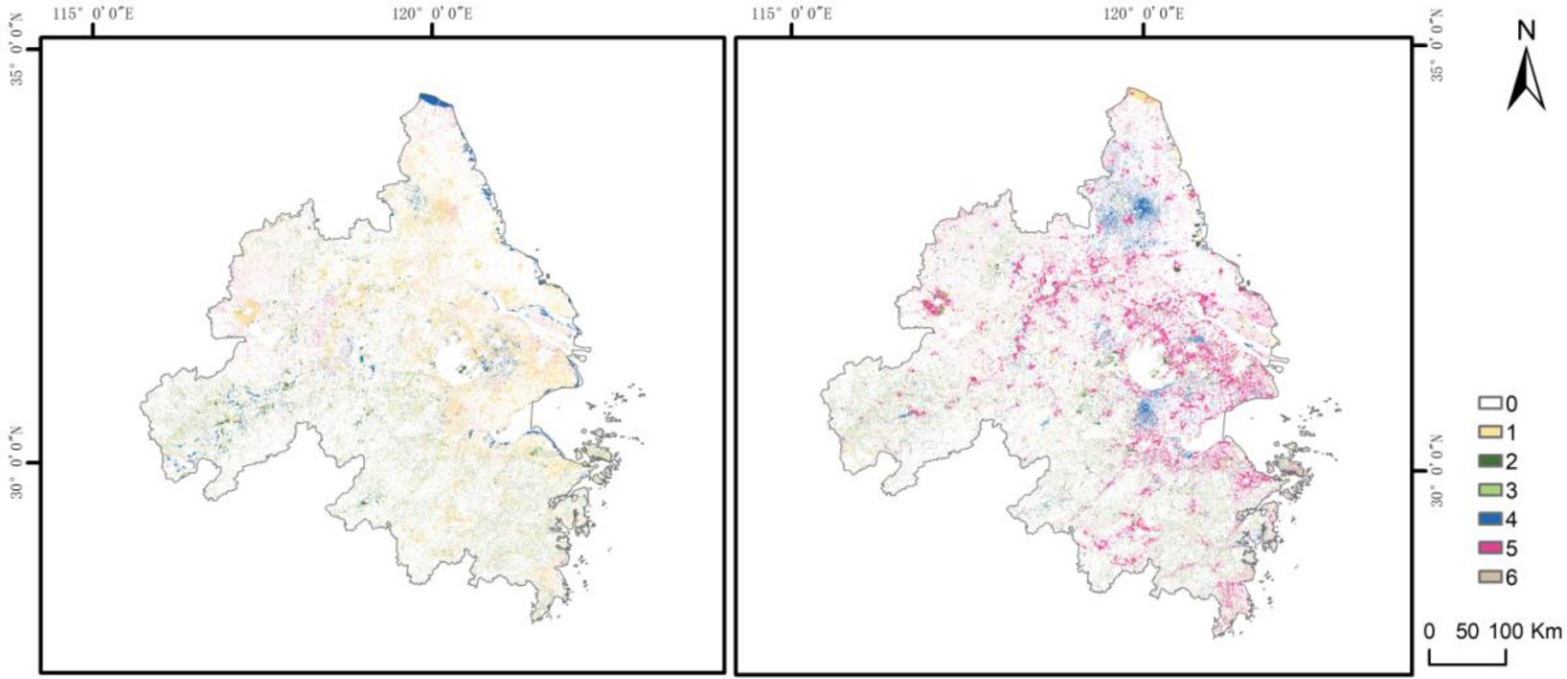
Land use downtrend (left) uptrend (right) in YRD region (0-unchanged).

Utilizing land use data between 2010 and 2020, various scenarios were established to simulate land use in 2030, as depicted in Fig 9. Under distinct scenarios, notable variations are observed in both land use demand and distribution. This is clearly illustrated in Table 7. In ND scenario, which considers only natural and anthropogenic factors without policy influence, the area of cropland is projected to be 92,685.90 km^2^, representing an 8.28% decrease compared to 2020. Both woodland and unused land show varying degrees of reduction, with decreases of 0.72% and 10.29% respectively. In contrast, developed land, watershed, and grassland exhibit increases, with growth rates of 28.37%, 8.69%, and 0.32%, respectively. Referring to Fig 10, the expansion of developed land extends outward from original land use, primarily occupying cropland and woodland. If left unrestricted, this expansion could pose threats to ecological environment and food security.

**Table 7:** Category area for various scenarios within YRD region for the year 2030 Unit:km^2^.

Type	ND scenario	EP scenario	CP scenario	UD scenario	BD scenario
Cropland	92,685.90	94,910.30	98,087.73	91,519.71	95,374.86
Woodland	54,671.98	55,379.77	54,788.76	54,495.87	54,863.73
Grassland	5,697.52	5,574.45	5,353.86	5,584.81	5,456.97
Watershed	22,589.06	22,690.72	22,805.31	22,438.04	22,879.04
Developed land	36,463.68	33,557.58	31,077.81	38,072.27	33,535.55
Unused land	27.31	21.54	21.08	24.76	24.18

In CP scenario, strict limitations on land conversion from cropland to other land types are implemented. In this particular scenario, projected cropland area in 2030 is estimated to reach 98,087.73 km^2^, indicating a 5.83% growth contrast with ND scenario. This highlights the potential efficacy of implementing measures aimed at protecting existing cropland and controlling the conversion of other land categories into agricultural use. Such actions can effectively safeguard agricultural land and ensure food security. By 2030, although developed land remains the most rapidly expanding land type, their expansion is reduced by 14.77% compared to ND scenario. Implementation of CP actions is expected to exert a limiting influence on the expansion of developed land, as indicated by this observation.

EP scenario demonstrates land use patterns like ND scenario. Compared to 2020, the current scenario demonstrates a decrease in cropland by 6.01 per cent and grassland by 1.85%, while woodland experiences a slight increase of 0.57%. An expansion of 9.18% is observed in the watersheds, with developed land expansion being the most noteworthy area of growth, although at a slower pace than ND scenario (18.14% rather than 28.37%).

In 2030 BD scenario, compared to 2030 ND scenario, cropland, woodland, and grassland all increase, with cropland having the largest increase of 2.90%, where cropland is relatively more strictly protected; ecological policies are strengthened to promote preservation and regeneration of woodland, with an increase of 0.35% in woodland. The decrease in grassland area, with a reduction rate of 4.22%, highlights the need for government to take appropriate measures to protect grasslands, prevent overgrazing and illegal exploitation. Policies should be formulated to promote sustainable management of grasslands and preserve the integrity of grassland ecosystems. These policies can facilitate sustainable management practices and ensure the ecological integrity of grassland ecosystems. The increase in watershed is 1.28%, effectively reducing the loss of watershed, which contributes to maintaining the health of the ecosystem. At the same time, it helps to avoid natural disasters that may result from excessive development, offering a more solid basis for the region’s sustainable economic growth. The most significant decrease of 18.28% in developed land resulted from the reduced likelihood of converting each category to developed land, while land use planning is enhanced to counteract the trend of excessive urbanization; It can be seen that there has been a reduction in the area of unused land, and this scenario requires avoiding over-exploitation and destruction of unused land, and government can provide incentives to encourage ecological restoration of unused land in order to reduce land degradation and protect ecological balance.

### 4.3 Analysis of carbon storage

**Fig 11:**
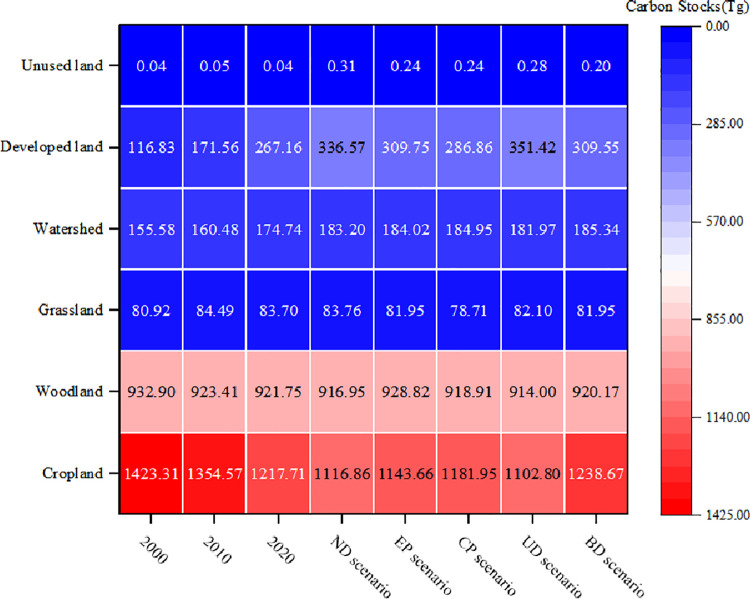
Carbon stocks in different land types under different scenarios.

Using LUCC maps and Invest model in conjunction with Eq (3), we are able to derive carbon storage within YRD region (Fig 11). In 2000, 2010, and 2020 was 2,709.577 Tg, 2,694.546 Tg, and 2,665.395 Tg respectively, exhibiting a declining trend over the years. Total reduction was 441.820 Tg, with an average annual reduction of 22.091 Tg. Specifically, between 2000 and 2010, carbon storage witnessed a reduction of 150.310 Tg, with a decrease rate of 0.55%. This accounted for 34% of the total reduction. From 2010 to 2020, carbon storage experienced a larger decline of 291.510 Tg, with a reduction rate of 1.08%. This took up 66% of aggregate reduction.

Carbon stock in 2030 fluctuates greatly under the scenarios of ND, CP, EP, UD and BD, with 2,637.645 Tg, 2,651.614 Tg, 2,648.447 Tg, 2,632.576 Tg and 2,735.878 Tg, respectively. In comparison to the carbon storage in 2020, carbon stocks of the first four land use scenarios have decreased. Notably, CP scenario exhibited the smallest fall, with a reduction of only 0.52%. The next is EP scenario. The CP scenario can help prevent the loss of carbon sinks, but conver cropland may lead to carbon release. However, with effective cropland management and agricultural practices, carbon emissions can be controlled, thereby supporting the dual carbon goals. The EP scenario helps an increase in forest cover and other carbon sinks, supporting the achievement of carbon neutrality. This scenario is closely aligned with the dual carbon targets and can effectively lower carbon emissions while enhancing carbon storage. Strengthening ecological protection fosters the health and stability of ecosystems, contributing to the long-term sustainable development of the YRD region. Under UD scenario, the greatest decline in carbon storage was observed, with a substantial decrease of 1.23%. This was primarily attributed to the continuous expansion of impermeable areas, which encroached upon cropland and other land types featured by high carbon density. This scenario is typically associated with higher carbon emissions, particularly in sectors like construction, transportation, and energy consumption. Therefore, it poses challenges in achieving carbon peak and carbon neutrality goals. Urbanization can cause ecological degradation and a reduction in carbon sink capacity. However, these negative effects can be partially mitigated through increased urban greening and improved energy efficiency. The 2030 BD scenario, on the other hand, shows an increase in carbon stock compared to 2020, with an increase of 2.64%. This scenario aims to optimize land use by reducing carbon emissions through effective planning and management while protecting carbon sinks. It is closely aligned with the dual carbon targets, supporting the achievement of carbon peak and carbon neutrality goals. The BD scenario fosters a win-win situation for economic growth and environmental protection, promoting sustainable development in the YRD region. Overall, the EP and BD scenarios are the most compatible with China’s dual carbon targets and can effectively support the carbon neutrality goals. In contrast, the ND and UD scenarios may lead to increased carbon emissions and provide limited support in achieving the dual carbon targets. While, the CP scenario supports carbon goals to some extent, it necessitates a balance between agricultural and ecological protection needs. Choosing appropriate scenarios and strategies will significantly influence the sustainable development of the YRD region.

**Fig 12:**
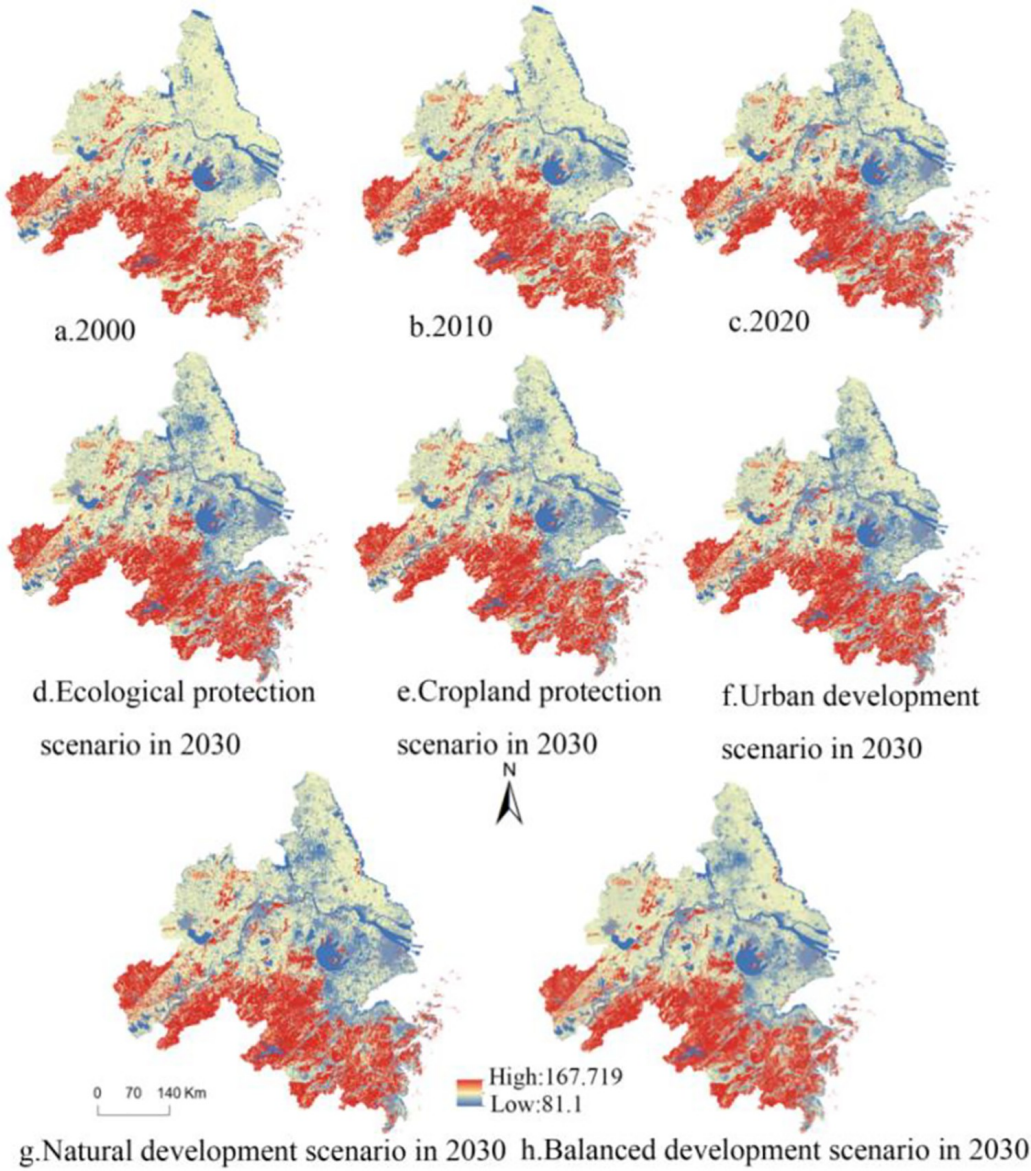
Carbon stocks in YRD region under different scenarios.

As shown in Fig 12, it can be observed that carbon stock changes between 2000 and 2010 mainly occurred in regions such as Shanghai, the northeastern part of Zhejiang Province, and the southern part of Jiangsu Province. From 2010 to 2020, throughout the region of study, sporadic fluctuations in carbon stock were noted, with a considerable decline in certain areas outweighing any increases. Regions which exhibited declining carbon stock were gathered in the southern Jiangsu Province, the northern Zhejiang Province, Shanghai, as well as the central Anhui Province. Spatial distribution of carbon stock reduction from 2000 to 2020 exhibited an "East more, West less" trend. With regards to spatial distribution, the carbon stock variation across YRD region was modest, exhibiting a mode of "North is high, South is low". To the north of Yangtze River, cropland, developed land, and watersheds were dominant land cover types, which contributed to lower carbon stock values, with the minimum value being 81.1 t/ha. In contrast, to the south of Yangtze River, woodland and grassland were primary land cover types, resulting in higher carbon stock values, with the maximum value reaching 167.719 t/ha.

**Fig 13:**
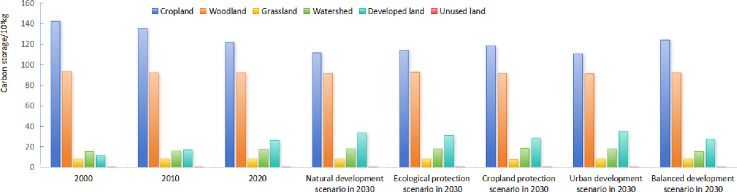
Carbon stocks in various land use types in YRD region, 2000–2030.

From Fig 13, it will be seen that carbon storage of cropland is consistently the highest in YRD region, with woodland ranking second. Carbon storage of only these two land types of accounts for over 80%. Dominance of plains characterized by extensive cropland and woodland with higher carbon storage per unit area explains the relatively higher carbon stock values observed across YRD region. Over the study period, there were minor variations in the proportion of carbon storage across various land types, except for a slight grow in developed and unused land. In contrast, there was no discernible trend of rising or falling in carbon storage proportion of other types, but rather a fluctuating change.

## 5 Discussion

### 5.1 Advantage and disadvantage of the model

To address the shortcomings of a single model, this paper integrates PLUS model with InVEST model to quantitatively evaluate land use changes and their impacts on carbon storage under different future development scenarios driven by policies. The coupling of models compensates the limitations of individual models, effectively harnessing the strengths of the PLUS model for simulating future land changes quantitatively and spatially, alongside the capabilities of the InVEST model in predicting carbon storage. Compared to the previous studies [40, 49], this paper simulates a relatively large number of future development scenarios and their compatibility with the ‘dual carbon policy’. The sustainable development has been analyzed, providing a more comprehensive perspective for future land use planning and development in YRD region. PLUS model is capable of generating high-resolution land use change scenarios and can also comprehensively consider economic, social, and environmental factors that influence LUCC. Furthermore, PLUS model can simulate various scenarios and capture the dynamic process of LUCC, providing time series data. However, the model’s parameters and settings require precise calibration [49]. Otherwise, they may affect the reliability of the simulation results. It also requires a large amount of high-quality input data, including driving factor data and land use data, which may lead to inaccurate model outputs in cases of incomplete data. PLUS model predicts future land demand based on past land expansion trends, which sets fixed conversion rules for each LUCC type [50]. These rules may change in the real scenarios due to future policies and planning. Additionally, the scenario settings in this study were generated with the references to other research although they did not incorporate national spatial planning. InVEST model can comprehensively assess changes in ecosystem services and include multiple service functions and values of ecosystems. The model’s interface is relatively friendly and well-suited for quick assessments and decision support. It supports the assessment of various ecosystem services, such as carbon storage, water quality, and soil retention, which can provide multi-dimensional analyses of ecosystem services. However, the carbon density data of various LUCC types in YRD region in this study were obtained from different studies. One of the limitations of both PLUS and InVEST models is the reliance on accurate and high-resolution input data. For instance, while the models use remote sensing data for land use/land cover classification, variations in image resolution and potential misclassifications can introduce errors in the simulations. Moreover, the temporal gaps between the available datasets can affect the model’s ability to predict future changes because it may not fully capture abrupt or non-linear land use transitions. Both PLUS and InVEST models are sensitive to the selection and calibration of various parameters, such as land use conversion rates, land retention probabilities, and driving factors like population density and GDP. Small variations in these parameters can lead to significant changes in the model outputs. For example, in the PLUS model, the conversion coefficients used for different land types can strongly influence the simulated land use patterns. Likewise, InVEST model’s results can be highly sensitive to the assumptions made on ecosystem service values and the spatial distribution of ecosystem types [49, 50]. In addition, carbon density data for various LUCC types in YRD region are sourced from disparate research [61, 62], which may cause inaccuracies in the estimation results due to variations in the selected indicators. Finally, it is important to acknowledge that InVEST model only considers carbon densities of various land use and land cover types, without accounting for the effect of vegetation types and their growth [67].

### 5.2 Impact of LUCC on carbon storage

The relationship between land use change on carbon storage is complex and dynamic. By conducting multi-scenario simulations using PLUS-InVEST model, we can gain a deeper understanding into the specific impacts of LUCC on carbon storage in the YRD region. This study provides scientific evidence for formulating policies related to ecological protection, land management, and sustainable development. Such research not only contribute to reducing greenhouse gas emissions but also help address the challenges posed by climate change. In contrast to previous studies [62, 66, 68], our study uniquely combines the PLUS and InVEST models to provide spatially explicit land use projections and carbon storage assessments under multiple policy scenarios, offering actionable insights for balancing development with ecological protection and mitigating the environmental impacts of urbanization in the YRD region. In accordance with simulations and predictions, it can be found that the prevailing trend of land use transition involves a shift from LUCC types with high carbon density to those with low. Consequently, this transition leads to a decline in total carbon storage within the region [15]. Implementation of CP actions has ensured economic benefits to some extent but has led to the loss of ecological benefits. EP scenarios can facilitate natural succession of woodland and grassland, playing an important role in restoring ecosystem carbon storage. However, cropland still does not receive effective protection. Under the BD scenario, it can be clearly seen that carbon stock has increased, which meets objectives of economic growth and environmental conservation. This scenario represents a pathway that meets both economic development goals and carbon sequestration objectives. In the future, policymakers and managers may consider developing under the BD scenario in response to China’s "dual-carbon" policy [56]. Carbon storage capacity varies among different land use types. Woodland serve as important carbon sinks [69], capable of absorbing carbon dioxide through photosynthesis and storing it as organic carbon. Research indicates that a decrease in cropland leads to a significant reduction in carbon storage [70]. When cropland is converted for food production or other agricultural uses, the release of soil carbon significantly increases. Agricultural practices such as tilling and fertilization also affect the organic carbon in the soil. Urbanization generally results in a decline in natural vegetation, resulting to reduced carbon storage [71]. Additionally, construction and development activities associated with urbanization release substantial amounts of carbon dioxide. By gradually transforming LUCC types with lower carbon density into those with higher carbon density, this situation can contribute to the restoration of carbon storage levels and align with the policy objectives of achieving carbon neutrality [72]. Policymakers should continue implementing policies that encourage the transformation of cropland into woodland and grassland, while simultaneously enhancing woodland protection measures and expanding woodland coverage. This approach represents a viable strategy for meeting the goals of carbon neutrality and ecosystem restoration [73, 74].

### 5.3 Limitations and prospects

Key policy factors, such as ecological red lines and urban development boundaries, are not incorporated into the model presented in this paper, which may restrict its applicability in real world scenarios. In addition, to increase the relevance of the study, global climate and socio-economic pathways, among other factors, could be incorporated into the land use simulation [75]. Spatial-temporal heterogeneity is used to understand multiscale ecological and environmental dynamics behind ecosystem change patterns. Understanding specific location and universal system index patterns is crucial to comprehend how environment varies across spatial, temporal, and biological scales. Therefore, distribution of ecosystem change patterns can be analyzed as a function of environmental variables, which can help identify both universal and site-specific attractors [76]. However, this article does not address this aspect. Future research in this focus could incorporate this aspect and include indices that show how spatial factors change over time to deepen the study. Additionally, analyzing sensitivity and uncertainty of the entire spatial domain, which is related to factors causing ecosystem changes and LUCC changes, would be beneficial [77]. Future studies could focus on improving the accuracy of land use and carbon storage projections by incorporating more recent, high-resolution data. As land use and environmental conditions evolve, using the latest remote sensing imagery and socio-economic datasets will enhance model precision. Integrating artificial intelligence and remote sensing technologies for real-time monitoring will also help track LUCC and carbon storage dynamics more effectively, reducing prediction errors and optimizing model parameters [78]. While our study focused on five key development scenarios, future research could explore additional scenarios, such as "climate adaptation" or "agriculture intensification," to gain a better understanding of long-term land use sustainability. Including scenarios with varying climate change impacts or alternative policy interventions (e.g., carbon trading or sustainable agriculture incentives) would provide a more comprehensive view of land-use dynamics in the region. Additionally, investigating the effects of climate change on land use and carbon storage, particularly in relation to extreme events like floods or droughts, could offer valuable insights on the resilience of land use systems. Understanding how these climate factors influence carbon storage will be crucial for adapting to changing environmental conditions. Furthermore, long-term research is needed to examine the sustained impacts of LUCC on carbon storage, particularly on how the carbon sink capacity of ecosystems evolves over time. Such research could provide critical evidence of developing more effective land management policies [79]. Lastly, incorporating dynamic models that account for different temporal and spatial scales will improve predictions of LUCC impacts. These models, integrating ecological, socio-economic, and climate factors, would enable a more comprehensive assessment of future scenarios [34].

Study findings present valuable insights into alterations observed in land use and carbon storage within YRD area across various scenarios encompassing CP, EP, ND, UD and BD until 2030. As such, these outcomes serve as important references for informing future national spatial planning endeavors and formulating sustainable development policies [66]. Government and policymakers can optimize carbon storage and ecological protection through comprehensive land use planning, balancing urban development, agriculture, and ecological conservation to meet carbon neutrality goals in the YRD region. In urban development, promoting green building designs and low-carbon transportation systems can reduce emissions. Specific steps include encouraging green building certifications and low-carbon materials, investing in electric vehicle networks, public transit, and cycling infrastructure, strengthening ecological protection by expanding nature reserves and restoring ecosystems. Key actions include expanding reserves in carbon-rich ecosystems like wetlands, forests, and grasslands. Governments should implement ecological restoration projects to recover natural carbon storage. They should strengthen biodiversity protection laws and maintain ecological corridors. Balancing agriculture with ecological protection can mitigate negative impacts. Policymakers should support sustainable agricultural practices, such as agroforestry and organic farming, and prevent the conversion of carbon-rich farmland to urban use. Promoting green infrastructure in both urban and rural areas will boost carbon storage. Specific actions include integrating green spaces, such as urban forests and green roofs, enhancing rural green infrastructure to improve soil health and carbon retention, and increasing urban tree cover while creating green belts [80, 81]. These strategies will balance urban growth, carbon sequestration, and ecological protection, supporting sustainable development in the YRD region and helping achieve China’s "dual-carbon" goals.

## 6 Conclusion

Based above analysis, the primarily conclusions as follows:

Over the period 2000–2020, there was a decline in the extent of cropland and woodland. Meanwhile, developed land has expanded significantly due to urbanization. There was a distinct spatial pattern found in carbon storage, featured by lower in the north and higher in the south, and there was no notable concentration of carbon storage changes in entire research areas and carbon storage decreased year by year. Throughout the entire duration of our study, depletion of carbon storage demonstrated a distinct spatial pattern featured by upper losses in the east and lesser losses in the west. By 2030, under the first four scenarios, carbon stock in YRD region declines to varying degrees. Notably, the UD scenario showcased the most substantial reduction in carbon storage, whereas CP scenario demonstrated the smallest decrease. Under the fifth scenario (BD), carbon stock has increased, and government may consider implementing corresponding policies and measures in the future to move towards BD scenario as far as possible. Woodland and cropland proved to be the main LUCC types for carbon sequestration in YRD region. Therefore, it is imperative to enhance woodland coverage to effectively augment carbon sequestration potential of YRD region. Decision-makers can consider economic development, ecological protection, and carbon storage when formulating balanced land use plans. They should aim to develop integrated land use strategies that combine urban construction, agricultural protection, and ecological preservation to promote coordinated development across all sectors. Moreover, relevant managers should actively pursue the construction of green infrastructure by applying ecological design principles to enhance the quality of urban and rural environments. Furthermore, the government can facilitate the implementation of dynamic adjustment mechanisms that allow for modifications to land use policies based on real-time changes in the environment and economy.

These recommendations and policies are designed to optimize land use planning through specific measures, promote sustainable development, and prioritize carbon storage. By implementing these suggestions, it is possible to achieve coordinated economic, ecological, and social development in the YRD region, thereby enhancing regional sustainability and ecological security. Future research should focus on further exploring the integration of socio-economic models, real-time data, and advanced technologies to improve the accuracy of carbon storage estimates and support long-term sustainability goals.

## Supporting information

S1 Dataset Minimal data set. (ZIP)

## References

[1] Lu X. H., Jiang H., Zhang X. Y., Jin J. X. Relationship between nitrogen deposition and LUCC and its impact on terrestrial ecosystem carbon budgets in China. Science China-Earth Sciences 2016,59,12. doi: 10.1007/s11430-015-5277-0

[2] Zhang T., Xin X., He F., Wang X. L., Chen K. How to promote sustainable land use in Hangzhou Bay, China? A decision framework based on fuzzy multiobjective optimization and spatial simulation. Journal of Cleaner Production 2023, 414, 137576. doi: 10.1016/j.jclepro.2023.137576

[3] Xu W. B., Xu H. Z., Li X. Y., Qiu H., Wang Z. Y. Ecosystem services response to future land use/cover change (LUCC) under multiple scenarios: A case study of the Beijing-Tianjin-Hebei (BTH) region, China. Technological Forecasting and Social Change 2024, 205. doi: 10.1016/j.techfore.2024.123525

[4] He X. Y., Liang J., Zeng G. M., Yuan Y. J., Li X. D. The Effects of Interaction between Climate Change and Land-Use/Cover Change on Biodiversity-Related Ecosystem Services. Global Challenges 2019, 3, 9. doi: 10.1002/gch2.201800095 31565394 PMC6733396

[5] Feng H. H., Wang S., Zou B., Nie Y. F., Ye S. C., Ding Y., et al. Land use and cover change (LUCC) impacts on Earth’s eco-environments: Research progress and prospects. Advances in Space Research 2023, 71, 3, 1418–1435. doi: 10.1016/j.asr.2022.09.054

[6] Wang W. J., Song H. Q., Min R. Q., Wang Q. F., Qi M. H. LUCC-induced dust aerosol change increase surface and reduce atmospheric direct radiative forcing in Northern China. Journal of Environmental Management 2024, 368. doi: 10.1016/j.jenvman.2024.122185 39151337

[7] Zhang J., Ren M. X., Lu X., Li Y., Cao J. J. Effect of the Belt and Road Initiatives on Trade and Its Related LUCC and Ecosystem Services of Central Asian Nations. Land 2022, 11, 6, 828.8. doi: 10.3390/land11060828

[8] Deng C. X., Liu J. Y., Liu Y. J., Li Z. W., Nie X. D., Hu X. Q., et al. Spatiotemporal dislocation of urbanization and ecological construction increased the ecosystem service supply and demand imbalance. Journal of Environmental Management 2021, 288, 15. doi: 10.1016/j.jenvman.2021.112478 33823451

[9] Gao H. R., Gong J., Liu J. K., Ye T. Effects of land use/cover changes on soil organic carbon stocks in Qinghai-Tibet plateau: A comparative analysis of different ecological functional areas based on machine learning methods and soil carbon pool data. Journal of Cleaner Production 2024, 434. doi: 10.1016/j.jclepro.2023.139854

[10] Mendoza-Ponce A., Corona-Núñez R., Kraxner F., Leduc S., Patrizio P. Identifying effects of land use cover changes and climate change on terrestrial ecosystems and carbon stocks in Mexico. Global Environmental Change-Human and Policy Dimensions 2018, 53, 12–23. doi: 10.1016/j.gloenvcha.2018.08.004

[11] Haszeldine R. S., Flude S., Johnson G., Scott V. Negative emissions technologies and carbon capture and storage to achieve the Paris Agreement commitments. Philosophical Transactions of the Royal Society A-Mathematical Physical and Engineering Sciences 2018, A 376: 20160447. doi: 10.1098/rsta.2016.0447 29610379 PMC5897820

[12] Wang Q. Z., Guan Q. Y., Sun Y. F., Du Q. Q., Xiao X., Luo H. P., et al. Simulation of future land use/cover change (LUCC) in typical watersheds of arid regions under multiple scenarios. Journal of Environmental Management, 2023,335. doi: 10.1016/j.jenvman.2023.117543 36848808

[13] Zaehle S., Bondeau A., Carter T. R., Cramer W., Erhard M., Prentice I. C., et al. Projected Changes in Terrestrial Carbon Storage in Europe under Climate and Land-use Change, 1990–2100. Ecosystems 2007,10, 380–401. doi: 10.1007/s10021-007-9028-9

[14] Foley J. A., DeFries R., Asner G.P., Barford C., Bonan G., Carpenter S. R., et al. Global Consequences of Land Use. Science, 2005,309, 570–574. doi: 10.1126/science.1111772 16040698

[15] Zhu L. Y., Song R. X., Sun S., Li Y., Hu K. Land use/land cover change and its impact on ecosystem carbon storage in coastal areas of China from 1980 to 2050. Ecological Indicators 2022,142,109178. doi: 10.1016/j.ecolind.2022.109178

[16] Li L., Huang X. J., Yang H. Optimizing land use patterns to improve the contribution of land use planning to carbon neutrality target. Land Use Policy 2024, 135. doi: 10.1016/j.landusepol.2023.106959

[17] Wei Q. Q., Abudureheman M., Halike A., Yao K. X., Yao L., Tang H., et al. Temporal and spatial variation analysis of habitat quality on the PLUS-InVEST model for Ebinur Lake Basin, China. Ecological Indicators 2022,145,109632. doi: 10.1016/j.ecolind.2022.109632

[18] Fu K. X., Chen L. X., Yu X. X., Jia G. D. How has carbon storage changed in the Yili-Tianshan region over the past three decades and into the future? What has driven it to change? Science of The Total Environment, 2024, 945, 174005. doi: 10.1016/j.scitotenv.2024.174005 38889815

[19] Fujisaki K., Perrin A. S., Garric B., Balesdent J., Brossard M. Soil organic carbon changes after deforestation and agrosystem establishment in Amazonia: An assessment by diachronic approach. Agriculture Ecosystems &Environment 2017, 245, 63–73. doi: 10.1016/j.agee.2017.05.011

[20] Zhu W. B., Zhang J. J., Cui Y. P., Zhu L. Q. Ecosystem carbon storage under different scenarios of land use change in Qihe catchment, China. Journal of Geographical Sciences 2022,30, 1507–1522. doi: 10.1007/s11442-020-1796-6

[21] Zhang M., Huang X. J., Chuai X. W., Yang H., Lai L., Tan J. Z. Impact of land use type conversion on carbon storage in terrestrial ecosystems of China: A spatial-temporal perspective. Scientific Reports 2015,5,10233. doi: 10.1038/srep10233 25975282 PMC4432567

[22] Han S. M., Jing Y. D., Liu Y. C. Simulation of land use landscape pattern evolution from a multi-scenario simulation: a case study of Nansi Lake Basin in China, Environmental Monitoring and Assessment, 2023,195:830. doi: 10.1007/s10661-023-11416-1 37296272

[23] Dang A. N., Kawasaki A. Integrating biophysical and socio-economic factors for land-use and land-cover change projection in agricultural economic regions. Ecological Modelling 2017, 344, 29–37. doi: 10.1016/j.ecolmodel.2016.11.004

[24] Vadrevu K. P., Ohara T. Focus on land use cover changes and environmental impacts in South/Southeast Asia. Environmental Research Letters 2020, 15, 10. doi: 10.1088/1748-9326/abb5cb

[25] Zhai R. T., Zhang C. R., Li W. D., Zhang X., Li X. K. Evaluation of Driving Forces of Land Use and Land Cover Change in New England Area by a Mixed Method. ISPRS International Journal of Geo-Information 2020, 9, 6. doi: 10.3390/ijgi9060350

[26] Zuo Y. T., Cheng J., Fu M. C. Analysis of Land Use Change and the Role of Policy Dimensions in Ecologically Complex Areas: A Case Study in Chongqing. Land 2022, 11, 5. doi: 10.3390/land11050627

[27] Quintero-Gallego M. E., Quintero-Angel M., Vila-Ortega J. J. Exploring land use/land cover change and drivers in Andean mountains in Colombia: A case in rural Quindio. Science of the Total Environment 2018, 634, 1288–1299. doi: 10.1016/j.scitotenv.2018.03.359 29660880

[28] Zhang Y. B., Yang J. C., Wang D. Y., Wang J., Yu L. X., Yan F. Q., et al. An Integrated CNN Model for Reconstructing and Predicting Land Use/Cover Change: A Case Study of the Baicheng Area, Northeast China. Remote Sensing 2021, 13, 23. doi: 10.3390/rs13234846

[29] Geng J. C., Shen S., Cheng C. X., Dai K. X. A hybrid spatiotemporal convolution-based cellular automata model (ST-CA) for land-use/cover change simulation. International Journal of Applied Earth Observation and Geoinformation 2022, 110. doi: 10.1016/j.jag.2022.102789

[30] Wang C. Y., Zhang X., Yang W., Wang G. G., Zhao Z. Z., Liu X., et al. Landsat-8 to Sentinel-2 Satellite Imagery Super-Resolution-Based Multiscale Dilated Transformer Generative Adversarial Networks. Remote Sensing 2023, 15, 22. doi: 10.3390/rs15225272

[31] Mengue V. P., de Freitas M. W. D., Silva T., Fontana D. C., Scott F. C. LAND -USE and land-cover change processes in Pampa biome and relation with environmental and socioeconomic data. Applied Geography 2020, 125. doi: 10.1016/j.apgeog.2020.102342

[32] Gao Z. Q., Gao W., Jie Z. The study of urban sprawl and simulation based on remote sensing and CLUS model. Remote Sensing and Modeling of Ecosystems for Sustainability IV 2007, 66791A. doi: 10.1117/12.726943

[33] He Z. J., Wang X. B., Liang X., Wu L., Yao J. Integrating spatiotemporal co-evolution patterns of land types with cellular automata to enhance the reliability of land use projections. International Journal of Geographical Information Science, 2024, 38, 5, 956–980. doi: 10.1080/13658816.2024.2314575

[34] Zhang Z. R., Li X. M., Liu X. Y., Zhao K. X. Dynamic simulation and projection of land use change using system dynamics model in the Chinese Tianshan mountainous region, central Asia. Ecological Modelling 2024, 487, 110564. doi: 10.1016/j.ecolmodel.2023.110564

[35] Mathewos M., Lencha S. M., Tsegaye M. Land Use and Land Cover Change Assessment and Future Predictions in the Matenchose Watershed, Rift Valley Basin, Using CA-Markov Simulation. Land 2022,11,1632. doi: 10.3390/land11101632

[36] Yu X. R., Xiao J. T., Huang J. T., Huang K., Li Y. Y., Lin Y. Y., et al. Simulation of Land Use Based on Multiple Models in the Western Sichuan Plateau. Remote Sensing 2023,15,14. doi: 10.3390/rs15143629

[37] Liang X., Guan Q. F., Clarke K. C., Liu S. S., Wang B. Y., Yao Y. Understanding the drivers of sustainable land expansion using a patch-generating land use simulation (PLUS) model: A case study in Wuhan, China. Computers, Environment and Urban Systems 2021,85,101569. doi: 10.1016/j.compenvurbsys.2020.101569

[38] Gao L. N., Tao F., Liu R. R., Wang Z. L., Leng H. J., Zhou T. Multi-scenario simulation and ecological risk analysis of land use based on the PLUS model: A case study of Nanjing. Sustainable Cities and Society, 2022,85,104055. doi: 10.1016/j.scs.2022.104055

[39] Wang S., Zhang X. Y., Adhikari K., Roland B., Zhuang Q. L., Wang Z. C., et al. Predicting soil organic carbon stocks under future land use and climate change conditions in Northeast China. Environmental Impact Assessment Review 2023,103,107278. doi: 10.1016/j.eiar.2023.107278

[40] Li J., Wang J., Li L., Zhou C. Y., Niu Q., Zhang C. M. Impact of Land Use Change on Carbon Stock in the Dongting Lake Ecological Economic Zone. Journal of Ecology. 2022,41(6),1156–1165. (In Chinese) doi: 10.13292/j.1000-4890.202206.026

[41] Berardi D., Brzostek E., Blanc-Betes E., Davison B., DeLucia E. H., Hartman M. D., et al. 21st-century biogeochemical modeling: Challenges for Century-based models and where do we go from here? Global Change Biology 2020, 12, 10, 774–788. doi: 110.1111/gcbb.12730

[42] Liu H. L., Liu H. B., Lei Q. L., Zhai L. M., Wang H. Y., Zhang J. Z., et al. Using the DSSAT model to simulate wheat yield and soil organic carbon under a wheat-maize cropping system in the North China Plain. Journal of Integrative Agriculture 2017, 16, 10, 2300–2307. doi: 10.1016/S2095-3119(17)61678-2

[43] Ball K. R., Burke I. C., Collins D. P., Kruger C. E., Yorgey G. G. Digging deeper: Assessing the predictive power of common greenhouse gas accounting tools for soil carbon sequestration under organic amendment. Journal of Cleaner Production 2023, 429. doi: 10.1016/j.jclepro.2023.139448

[44] Zhang Y. C., Liu X. J., Lei L. P., Liu L. Y. Estimating Global Anthropogenic CO2 Gridded Emissions Using a Data-Driven Stacked Random Forest Regression Model. Remote Sensing 2022, 14, 16. doi: 10.3390/rs14163899

[45] Schaefer K., Collatz G. J., Tans P., Denning A. S., Baker I., Berry J., et al. Combined Simple Biosphere/Carnegie-Ames-Stanford Approach terrestrial carbon cycle model. Journal of Geophysical Research-Bbiogeosciences 2008, 113, G3. doi: I10.1029/2007JG000603

[46] Babbar D., Areendran G., Sahana M., Sarma L., Raj K., Sivadas A. Assessment and prediction of carbon sequestration using Markov chain and InVEST model in Sariska Tiger Reserve, India. Journal of Cleaner Production, 2020,278,123333. doi: 10.1016/j.jclepro.2020.123333

[47] Guo W., Teng Y. J., Li J., Yan Y. G., Zhao C.W., Li Y. X., et al. A new assessment framework to forecast land use and carbon storage under different SSP-RCP scenarios in China. Science of the Total Environment. 2024, 912, 169088. doi: 10.1016/j.scitotenv.2023.169088 38056670

[48] Scordo F., Lavender T. M., Seitz C., Perillo V. L., Rusak J. A., Piccolo M. C., et al. Modeling Water Yield: Assessing the Role of Site and Region-Specific Attributes in Determining Model Performance of the InVEST Seasonal Water Yield Model. Water 2018,10,11. doi: 10.3390/w10111496

[49] Li P. C., Chen J. D., Li Y. X., Wu W. Using the InVEST-PLUS Model to Predict and Analyze the Pattern of Ecosystem Carbon storage in Liaoning Province, China. Remote Sensing. 2023, 15, 4050. doi: 10.3390/rs15164050

[50] Li L., Ji G. X., Li Q. S., Zhang J. C., Guo H. S., Jia M. Y., et al. Spatiotemporal Evolution and Prediction of Ecosystem Carbon Storage in the YiluoRiver Basin Based on the PLUS-InVEST Model. Forests, 2023, 14(12), 2442. doi: 10.3390/f14122442

[51] Gao J., Wang L. C. Embedding spatiotemporal changes in carbon storage into urban agglomeration ecosystem management—A case study of the Yangtze River Delta, China. Journal of Cleaner Production 2019, 237, 117764. doi: 10.1016/j.jclepro.2019.117764

[52] Zhu W. Z., Dong W., Qin G. W. Yang Y. J. Coordinated carbon reduction mechanism and policy design to achieve carbon peak and neutrality goals in the Yangtze River Delta. Sustainable Energy Technologies and Assessments 2023, 56, 103113. doi: 10.1016/j.seta.2023.103113

[53] Chuai X. W., Huang X. J., Wang W. J., Zhao R. Q., Zhang M., Wu C. Y. Land use, total carbon emissions change and low carbon land management in Coastal Jiangsu, China. Journal of Cleaner Production 2015, 103, 77–86. doi: 10.1016/j.jclepro.2014.03.046

[54] Qiao W., Huang X. The impact of land urbanization on ecosystem health in the YRD urban agglomerations, China. Cities 2022,130,103981. doi: 10.1016/j.cities.2022.103981

[55] Yin R., Li X., Fang B. The Relationship between the Spatial and Temporal Evolution of Land Use Function and the Level of Economic and Social Development in the YRD. International Journal of Environmental Research and Public Health 2023, 20, 2461. doi: 10.3390/%20ijerph20032461 36767830 PMC9916072

[56] Li W., Chen Z. J., Li M. C., Zhang H., Li M. Y., Qiu X. Q., et al. Carbon emission and economic development trade-offs for optimizing land-use allocation in the Yangtze River Delta, China. Ecological Indicators, 2023,147,109950. doi: 10.1016/j.ecolind.2023.109950

[57] Huang Y., Wu J. Spatial and temporal driving mechanisms of ecosystem service trade-off/synergy in national key urban agglomerations: A case study of the YRD urban agglomeration in China. Ecological Indicators 2023,154,110800. doi: 10.1016/j.ecolind.2023.110800

[58] Liu C. G., Sun W., Li P. X., Zhang L. C., Li M. Differential characteristics of carbon emission efficiency and coordinated emission reduction pathways under different stages of economic development: Evidence from the YRD, China. Journal of Environmental Management 2023,330,117018. doi: 10.1016/j.jenvman.2022.117018 36586363

[59] Luo D., Liang L. W., Wang Z. B., Chen L. K., Zhang F. Exploration of coupling effects in the Economy–Society–Environment system in urban areas: Case study of the Yangtze River Delta Urban Agglomeration. Ecological Indicators 2021,128,107858. doi: 10.1016/j.ecolind.2021.107858

[60] Wang Q. Y. Research on Ecosystem Service Assessment and Value Realization Mechanism in Yangtze River Delta. Nanjing Forestry University, 2022. (In Chinese) doi: 10.27242/d.cnki.gnjlu.2022.000125

[61] Zhang C., Tian H. Q., Chen G. S., Chappelka A., Xu X. F., Ren W., et al. Impacts of urbanization on carbon balance in terrestrial ecosystems of the Southern United States. Environmental pollution 2012,164,89–101. doi: 10.1016/j.envpol.2012.01.020 22343525

[62] Zhou J., Zhang X. R., Mu F. Y., Zhao R. Y., Zhou W., Li M. M. Research on the Spatial Pattern Reconstruction of Soil Organic Carbon Stock Based on CA-Markov Model: A Case Study of the Pan YRD Area. Resources and Environment in the Yangtze Basin. 2018,27(7),1565–1575. (in Chinese) doi: 10.11870/cjlyzyyhj201807016

[63] Wang K. L., Bian Y. J., Cheng Y. H. Exploring the Spatial Correlation Network Structure of Green Innovation Efficiency in the Yangtze River Delta, China, Sustainability, 2022,14(7), 3903. doi: 10.3390/su14073903

[64] Shi G., Jiang N., Yao L. Q. Land Use and Cover Change during the Rapid Economic Growth Period from 1990 to 2010: A Case Study of Shanghai. Sustainability, 2018,10(2),426–426. doi: 10.3390/su10020426

[65] Zheng X. Q., Zhao L., Xiang W. N., Li N., Lv L. N., Yang X. A coupled model for simulating spatio-temporal dynamics of land-use change: A case study in Changqing, Jinan, China. Landscape and Urban Planning 2012,106,1.10. doi: 1016/j.landurbplan.2012.02.006

[66] Zhang D., Wang X. R., Qu L. P., Li S. C., Lin Y. P., Yao R., et al. Land use/cover predictions incorporating ecological security for the Yangtze River Delta region, China. Ecological Indicators 2020,119,106841. doi: 10.1016/j.ecolind.2020.106841

[67] Wei B. H., Kasimu A., Reheman R., Zhang X. L., Zhao Y. Y., Aizizi Y., et al. Spatiotemporal characteristics and prediction of carbon emissions/absorption from land use change in the urban agglomeration on the northern slope of the Tianshan Mountains. Ecological Indicators 2023,151,110329. doi: 10.1016/j.ecolind.2023.110329

[68] Zhao Y. H., Su D., Bao Y., Yang W., Sun Y. B. A CLUMondo Model-Based Multi-Scenario Land-Use Change Simulation in the Yangtze River Delta Urban Agglomeration, China. Sustainability 2022, 14, 15336. doi: 10.3390/su142215336

[69] Ge J. M., Lin B. Q. Convergence or divergence? Unraveling the global development pattern of forest carbon sink. Environmental Impact Assessment Review 2024, 105, 107442. doi: 10.1016/j.eiar.2024.107442

[70] Ma D. L., Huang Q. J., Wang Q., Lin Z. X., Xu H. L. Simulation of LUCC Scenarios and Analysis of the Driving Force of Carbon Stock Supply Changes in the North China Plain in the Context of Urbanization. Forests 2024, 15, 8, 1414. doi: 10.3390/f15081414

[71] Borges E. C., Paz I., Neto A. D. L., Willinger B., Ichiba A., Gires A., et al. Evaluation of the spatial variability of ecosystem services and natural capital: the urban land cover change impacts on carbon stocks. International Journal of Sustainable Development and World Ecology 2021, 28, 4, 339–349. doi: 10.1080/13504509.2020.1817810

[72] Deng C. X., Peng Y., Li K., Li Z. W. Watershed Land Use Transformation and Ecological Environmental Effects based on Multi-Scenario Simulation of Production-Ecology-Living Space. Chinese Journal of Ecology, 2021,40(08),2506–2516. doi: 10.13292/j.1000-4890.202108.031

[73] Li X., Chen Y. M. Projecting the future impact of China’s cropland balance policy on ecosystem services under the shared socioeconomic pathways. Journal of Cleaner Production, 2020,250,119489. doi: 10.1016/j.jclepro.2019.119489

[74] Xu C. L., Zhang Q. B., Yu Q., Wang J. P., Wang F., Qiu S. et al. Effects of land use/cover change on carbon storage between 2000 and 2040 in the Yellow River Basin, China. Ecological Indicators, 2023,151,110345. doi: 10.1016/j.ecolind.2023.110345

[75] Chen Q. B., Ning Y. Projecting LUCC dynamics and ecosystem services in an emerging urban agglomeration under SSP-RCP scenarios and their management implications. Science of the Total Environment 2024, 949, 175100. doi: 10.1016/j.scitotenv.2024.175100 39084394

[76] Levin S. A. The problem of pattern and scale in ecology: the Robert H. MacArthur award lecture. Ecology, 1992, 1. doi: 10.2307/1941447

[77] Hoque M. A., Cui S. H., Islam I., Xu L. L., Ding S. P. Dynamics of plantation forest development and ecosystem carbon storage change in coastal Bangladesh. Ecological Indicators, 2021, 130, 107954. doi: 10.1016/j.ecolind.2021.107954

[78] Liu Y. S., Huang X. X., Liu Y. Q. Detection of long-term land use and ecosystem services dynamics in the Loess Hilly-Gully region based on artificial intelligence and multiple models. Journal of Cleaner Production 2024, 447, 141560. doi: 10.1016/j.jclepro.2024.141560

[79] Lyu J. Q., Fu X. H., Lu C., Zhang Y. Y., Luo P. P., Guo P., et al. Quantitative assessment of spatiotemporal dynamics in vegetation NPP, NEP and carbon sink capacity in the Weihe River Basin from 2001 to 2020. Journal of Cleaner Production 2023, 428, 139384. doi: 10.1016/j.jclepro.2023.139384

[80] Liu J. L., Zhu Y., Zhang Q., Cheng F. Y., Hu X., Cui X. H., et al. Transportation Carbon Emissions from a Perspective of Sustainable Development in Major Cities of Yangtze River Delta, China. Sustainability 2021, 13(1), 192. doi: 10.3390/su13010192

[81] Wu W., Zhang T. T., Xie X. M., Huang Z. Regional low carbon development pathways for the Yangtze River Delta region in China. Energy Policy 2021, 151, 112172. doi: 10.1016/j.enpol.2021.112172

